# Genome-wide identification, characterization, and genetic diversity of *CCR* gene family in *Dalbergia odorifera*

**DOI:** 10.3389/fpls.2022.1064262

**Published:** 2022-12-19

**Authors:** Yue Wang, Jieru Xu, Wenxiu Zhao, Jia Li, Jinhui Chen

**Affiliations:** ^1^ Hainan Yazhou Bay Seed Laboratory, School of Forestry, Sanya Nanfan Research Institute of Hainan University, Sanya, China; ^2^ Key Laboratory of Genetics and Germplasm Innovation of Tropical Special Forest Trees and Ornamental Plants, Ministry of Education/Engineering Research Center of Rare and Precious Tree Species in Hainan Province, School of Forestry, Hainan University, Haikou, China; ^3^ Research Institute of Forestry, Hainan Academy of Forestry (Hainan Academy of Mangrove), Haikou, China

**Keywords:** *Dalbergia odorifera*, cinnamoyl-CoA reductase, simple sequence repeat, genetic diversity, forest breeding

## Abstract

**Introduction:**

Lignin is a complex aromatic polymer plays major biological roles in maintaining the structure of plants and in defending them against biotic and abiotic stresses. Cinnamoyl-CoA reductase (CCR) is the first enzyme in the lignin-specific biosynthetic pathway, catalyzing the conversion of hydroxycinnamoyl-CoA into hydroxy cinnamaldehyde. *Dalbergia odorifera* T. Chen is a rare rosewood species for furniture, crafts and medicine. However, the *CCR* family genes in *D. odorifera* have not been identified, and their function in lignin biosynthesis remain uncertain.

**Methods and Results:**

Here, a total of 24 genes, with their complete domains were identified. Detailed sequence characterization and multiple sequence alignment revealed that the DoCCR protein sequences were relatively conserved. They were divided into three subfamilies and were unevenly distributed on 10 chromosomes. Phylogenetic analysis showed that seven DoCCRs were grouped together with functionally characterized CCRs of dicotyledons involved in developmental lignification. Synteny analysis showed that segmental and tandem duplications were crucial in the expansion of *CCR* family in *D. odorifera*, and purifying selection emerged as the main force driving these genes evolution. Cis-acting elements in the putative promoter regions of *DoCCRs* were mainly associated with stress, light, hormones, and growth/development. Further, analysis of expression profiles from the RNA-seq data showed distinct expression patterns of *DoCCRs* among different tissues and organs, as well as in response to stem wounding. Additionally, 74 simple sequence repeats (SSRs) were identified within 19 *DoCCRs*, located in the intron or untranslated regions (UTRs), and mononucleotide predominated. A pair of primers with high polymorphism and good interspecific generality was successfully developed from these SSRs, and 7 alleles were amplified in 105 wild *D. odorifera* trees from 17 areas covering its whole native distribution.

**Discussion:**

Overall, this study provides a basis for further functional dissection of *CCR* gene families, as well as breeding improvement for wood properties and stress resistance in *D. odorifera*.

## Introduction

*Dalbergia odorifera* T. Chen is a medium-sized evergreen tree that belongs to the Fabaceae family (Hong et al., 2020). The heartwood of *D. odorifera* is a superior material for manufacturing pricey furniture and exquisite crafts because of its distinctive pattern, solid structure, and pleasant aroma (Sun et al., 2015). In addition, it has long been known as “JiangXiang” in Chinese medicine and was frequently used to treat stasis, halt bleeding, and alleviate pain (Zhao et al., 2020). Owing to its high medicinal and commercial value, *D. odorifera* is quickly disappearing: only a limited number of trees are found in parts of their original habitat, as highly fragmented populations are now remaining in the forests of Hainan Island (China) (Liu et al., 2019a). Over 300 tonnes of raw *D. odorifera* heartwood are required annually, with a production value of over 700 million USD (Meng et al., 2019). However, *D. odorifera* is a slow-growing tree in its natural environment, taking 15 years to form heartwood (Zhao et al., 2020). Therefore, it is urgent to understand formation of heartwood and cultivate *D. odorifera* trees with good wood quality.

Lignin makes up around 20-30% of the mass of a wood and is the second-most prevalent biopolymer on Earth (after cellulose) (Leplé et al., 2007). The biosynthesis of monolignols (p-coumaryl, coniferyl, and sinapyl alcohols), which are the primary building blocks of lignin, requires the action of several enzymes. The cinnamoyl-CoA reductase (CCR; EC 1.2.1.44) is the first enzyme of the monolignol synthesis pathway, and belong to the short-chain dehydrogenase/reductase superfamily (SDR) (Goffner et al., 1994). It catalyzes the reduction of a variety of hydroxycinnamoyl CoA (commonly including p-coumaroyl-CoA and feruloyl-CoA) to its corresponding cinnamaldehyde, providing the precursor molecules for lignin formation. CCR enzymes are widely distributed in a variety of plants, mostly in the form of multigene families. The first *CCR* gene was characterized and cloned from *Eucalyptus gunnii* (Goffner et al., 1994), and since then *CCR* homologs have been investigated in diverse plant species, such as *Arabidopsis thaliana*, *Medicago truncatula*, *Oryza sativa* and *Populus trichoc*arpa (Giordano et al., 2014). Most modern land plants and all vascular plants are thought to contain at least one functional *CCR*, which is a must for any plant species with lignified tissues (Barakat et al., 2011). The majority of *CCR* homologs exhibit elevated levels of expression during development, particularly in stem, root, and xylem cells, which demand the lignin’s greater structural support (Barros et al., 2015). However, certain *CCRs* are not constitutively expressed throughout development and are only upregulated during enhanced lignification in response to stressors (Lauvergeat et al., 2001). Further functional studies of *CCR* family members in *A. thaliana* revealed that *AtCCR1* participates in lignification, whereas *AtCCR2* was mainly induced by abiotic or biotic stresses and was involved in plant defense mechanisms, and similar mechanisms have been reported in *Panicum virgatum* and *Zea mays*. In *A. thaliana* irregular xylem 4 (irx4) mutant, that does not contain *AtCCR1*, the xylem was collapsed, and the lignin content was decreased to 50% of the wild type (Goujon et al., 2003). In transgenic poplar, the down-regulation of *CCR* not only affected lignin content and the degree of lignification, it also reduced hemicellulose and pectin synthesis (Leplé et al., 2007). However, investigations on *CCR* genes in *D. odorifera* have been limited.

To comprehensively understand its putative roles in *D. odorifera*, a systematic analysis of the *CCR* gene family is needed at the genome level. As the whole-genome sequence of *D. odorifera* has now been made available (Hong et al., 2020), it provides an opportunity to execute such a task. Genome availability also facilitates the evaluation of genetic diversity based on simple sequence repeat (SSR) markers. In this study, all *CCR* family members were identified in *D. odorifera*. Subsequently, molecular characterization, chromosomal localization, gene structure, conserved domain, motif composition, evolution and cis-acting elements of *DoCCR* genes were systematically analyzed. Additionally, RNA-seq data was used to assess the transcriptional expression patterns of *DoCCRs* in various tissues and under wounding condition. Furthermore, we identified and characterized the SSR loci from *DoCCR* gene family, and evaluate SSR primers and polymorphisms in a set of wild *D. odorifera* samples from the Hainan Island. In general, this study lays a theoretical foundation for the study of *DoCCRs* gene function and provides a new perspective for genetic improvement in *D. odorifera* breeding.

## Materials and methods

### Identification and chromosome location of the *CCR* gene family

Genome and protein data of *D. odorifera* from the GigaScience Database (GigaDB, http://gigadb.org/dataset/100760) (Hong et al., 2020) and some protein sequences from our previous transcriptome projects (Zhao et al., 2022) were obtained as local database. In this study, two strategies were used to identify potential CCR family members in *D. odorifera*. First, we collected several experimentally validated CCR proteins from different species, such as by biochemical *in vitro* analysis or genetic modifications *in vivo* (**Table S1**). These sequences were used to build a family-specific hidden Markov model (HMM) profile against the protein database of *D. odorifera* using the HMMER software with an e-value threshold of 1e-10 (Finn et al., 2011). Second, BLASTP analyses was conducted where all the experimentally validated sequences and other potential CCRs retrieved from UniProt (https://www.uniprot.org/) were used as queries to predict CCRs in the protein database of *D. odorifera*. Additionally, the complete genome sequence was searched with TBLASTN to detect genes that could have been earlier misannotated, or were missed out by using the protein database. The e-value for both rounds of BLAST was set at 1e-5. With the help of PFAM (http://pfam.xfam.org/), SMART (http://smart.emblheidelberg.de/), CDD (https://www.ncbi.nlm.nih.gov/cdd/) and InterProScan (http://www.ebi.ac.uk/interpro/) databases, all the putative *D. odorifera* CCRs identified from the HMM and BLAST searches were accepted for further analyses only if they contained conserved CCR domain compared with each database. The last step involved manual annotation to resolve any discrepancy between wrongly predicted genes and their actual chromosomal positions. For *D. odorifera* short name gene nomenclature, we adopted a species-related prefix (Do) followed by the gene family abbreviation (CCR) (Carocha et al., 2015). According to their location on the 10 primary chromosomes, the *DoCCR* family members were assigned sequential numbers. These *DoCCR* genes were plotted according to their physical position in the 10 chromosome scaffolds of *D. odorifera* using TBtools (Chen et al., 2020).

### Physicochemical features and subcellular location prediction

The physicochemical properties of the DoCCR proteins, including the number of amino acids, molecular weight (MW), isoelectric point (pI), instability index (Ii), and grand average of hydropathicity (GRAVY) were analyzed with the help of an online ExPASy-ProtParam tool (http://web.expasy.org/protparam/) (Wilkins et al., 1999). The subcellular localization and signal peptide were predicted by using BUSCA (https://busca.biocomp.unibo.it/) (Savojardo et al., 2018) and SignalP (https://services.healthtech.dtu.dk/service.php?SignalP-5.0) (Almagro Armenteros et al., 2019), respectively.

### Conserved domain and gene structures analysis

The conserved motifs in full-length DoCCR proteins were predicted with the help of the online MEME tool (http://meme-suite.org/tools/meme) (Bailey et al., 2009), with the parameters of any number of repetitions, optimum motif width of 6-50 residues, and searching for 10 motifs. In addition, exon and intron positions were obtained from genome annotation files of *D. odorifera* (Hong et al., 2020). Wherever available, genes and their complementary DNA (cDNA) sequences were aligned to check the intron/exon structure. The software TBtools then was used to visualize the conserved motifs and gene structures (Chen et al., 2020).

### Multiple sequence alignment and phylogenetic analysis

Multiple sequence alignment of DoCCR proteins and functional CCR proteins identified from 19 plant species was performed using MUSCLE with default parameters (**Table S1**) (Edgar, 2004). The phylogenetic tree was constructed by MEGA11 software with the neighbor-joining (NJ) method and the parameters of Poisson model, 50% partial deletion and 1,000 bootstrap replicates (Saitou and Nei, 1987; Tamura et al., 2021). Then, the phylogenetic tree was visualized by the help of EvolView (https://www.evolgenius.info/evolview) (Subramanian et al., 2019). The other NJ tree was constructed using only DoCCR proteins with the same parameters.

### Gene duplication and synteny analysis

Genomes and gene annotation files of four selected species (*A. thaliana*, *M. truncatula*, *O. sativa* and *Z. mays*) were downloaded from Phytozome (https://phytozome.jgi.doe.gov/). Comparing the protein sequences of *D. odorifera* with themselves or those of other species using BLASTP with e-value of 1e-5. The BLASTP output file and a corresponding simplified gene annotation file (that contained chromosome, gene, start, and end) served as an input for Multiple Collinearity Scan toolkit (MCScanX) to identify syntenic blocks and distinct duplication events using default parameters (Wang et al., 2012). The nonsynonymous substitution (Ka) and synonymous substitution (Ks) rates of each duplicated *DoCCR* gene pair were calculated using KaKs_Calculator 2.0 (Wang et al., 2010).

### Promoter cis-acting element analysis and protein interaction network

The promoter sequences of all *DoCCR*s were submitted to PlantCARE (https://bioinformatics.psb.ugent.be/webtools/plantcare/html/) to analyze their cis-acting elements (Lescot et al., 2002). For this purpose, a 2,000 bp sequence upstream of the translation start site was taken to identify cis-acting elements. Moreover, STRING online service (http://string-db.org/) (Szklarczyk et al., 2021) was used to construct an interaction network, with homologous proteins from *A. thaliana*, to analyze the relationship of DoCCRs with other proteins.

### Expression pattern analysis of *DoCCRs*

To examine transcript accumulation patterns of *DoCCR* genes, the publicly available transcriptomics data of *D. odorifera* in various tissues (PRJNA552194 and PRJCA008328) and under wounding stress (PRJNA612155) were download from the National Center for Biotechnology Information (NCBI, https://www.ncbi.nlm.nih.gov/) or the National Genomics Data Center (NGDC, https://ngdc.cncb.ac.cn/). 10 different tissues used were as follows: flower, seed, leaf, root, stem, three regions of vascular cambium (Top VC: isolated from the one-year-old branch; Middle VC: isolated from the vascular cambium, 180cm off the ground; Bottom VC: isolated from the vascular cambium, 30cm off the ground), two regions of xylem (TZ: transition zone; SW: sapwood). Transcriptome data used to study wounding response were taken from discolored (D), and healthy (H) zones of the stem after three weeks of pruning (Sun et al., 2020). For RNA-seq datasets, adapters and low-quality sequences (including reads with unknown base pairs “N”) were removed from raw sequence reads using fastp software with the parameters of q = 20 and n = 15 (Chen et al., 2018). Then, the clean reads were mapped to *D. odorifera* genome with the help of HISAT2 software (Kim et al., 2019). The number of read for each gene was counted with the help of featureCounts by adapting default parameters (Liao et al., 2014). Transcripts per million (TPM) was used as an index to measure the expression level of genes.

### Plant materials and growth conditions

For SSR validation and diversity analysis in this study, a set of diverse materials from the natural population of *D.odorifera* in the Hainan Island were collected. The rules for sample collection were as follows: (i) collect material from each region in accordance with the geographic zoning of Hainan; (ii) collect old or famous trees of *D.odorifera* published in the available literature or public information; (iii) collect material with an age of more than 20 years and a diameter at breast height of more than 8 cm; (iv) collect material with a geographical distance of more than 10 km between different individuals; (v) collect material that avoids artificially propagated or introduced individuals as much as possible; (vi) collect material from each region with reference to the population size of *D.odorifera* in that region. A total of 105 *D. odorifera* samples were collected from 17 areas in Baoting, Baisha, Changjiang, Chengmai, Dingan, Dongfang, Danzhou, Haikou, Ledong, Lingao, Lingshui, Qionghai, Sanya, Tunchang, Wenchang, Wanning and Wuzhishan (**Table S2**). Young leaves were collected and desiccated by silica gel and stored at -80°C for later use. To investigate the interspecific generality of potential SSR loci in *D.odorifera*, three samples of *Dalbergia tonkinensis*, three samples of *Dalbergia sissoo* and one sample of *Dalbergia cochinchinensis* from the genus *Dalbergia* and three samples of *Pterocarpus santalinus* from the genus *Pterocarpus* were collected from seedling seed orchards in Dongfang and Sanya.

### SSR identification and primer design

The SSRs in the *DoCCR* gene sequences were detected by MISA program (Beier et al., 2017), and then their type and distribution characteristics were manually counted. The minimum repeat number for the unit size for mononucleotide to hexanucleotide were set to 10, 6, 5, 5, 5 and 5 respectively (Thiel et al., 2003). The obtained SSR molecular markers were screened, the repeat units were retained as dinucleotide to hexanucleotide, and the loci that were composed completely of C/G bases were removed. The Primer3 software (http://frodo.wi.mit.edu/primer3) (Untergasser et al., 2012) was used for primer (both forward and reverse) designing with parameters: product size 100-300 bp, primer size 18-25 bp, GC content 50-60% and melting temperature 56°C-62°C with 60°C as the optimum temperature. The specificity of each primer pairs in the genomic sequence was then checked by BLAST. One of the primers in the primer pair that met the criteria was elongated for the M13 (-21) 18 bp sequence at the 5′ end (5′-TGTAAAACGACGGCCAGT-3′) for economic fluorescent labelling (Schuelke, 2000).

### Validation of SSR markers

Total genomic DNA was extracted from 500 mg of leaf (from each tree sample) using the Plant Genome DNA Extraction Kit (DP305, Tiangen Biotech, Beijing, China) according to the instructions. The DNA samples were tested by 1% agarose gel electrophoresis for quality (clear bands, no RNA or protein contamination) and by microspectrophotometer for purity (1.9 ≤ A260/A280 ≤ 2.2, A260/A230 ≥ 2.0). The DNA samples were stored in an ultra-low temperature refrigerator at -80°C. Qualified DNA samples were used for subsequent experiments.

These newly developed SSR primer pairs were tested on 10 relatively geographically distant samples from 105 DNA samples of *D. odorifera*. Primer pairs with stable amplification results, no spurious peaks, good reproducibility and polymorphism were finally selected to genotype all samples. For PCR amplification, 15 μL reaction mixture was setup, which comprised of 2 × Taq PCR Master Mix 7.5 μL, mixed primer (with fluorescent group that can bind to M13 universal linker sequence) 2.0 μL, DNA template 1 μL (50-200 ng), ddH_2_O 4.5 μL. The amplification was performed as follows: 96°C for 3 min, 30 cycles of 96°C for 0.5 min, gradient temperatures (56-62°C) for 0.5 min, 72°C for 1 min, followed by a final extension of 72°C for 10 min, and 12°C preservation. Fragment analysis was performed on an 3730xl Genetic Analyzer (Applied Biosystems, MA, USA) using GeneScan 500 LIZ (Applied Biosystems, MA, USA) as a size standard. Microsatellite genotypes were examined using GeneMarker software (Hulce et al., 2011).

### Genetic diversity analysis

SSR loci were assessed for their value in the investigation of genetic diversity in *D. odorifera* population. The number of amplified microsatellite alleles (*Na*), the number of effective alleles (*Ne*), the observed heterozygosity (*Ho*), the expected heterozygosity (*He*), the shannon’s information index (*I/sHA*) and the fixation index (*F*) were calculated using GenAlEx 6.5 program (Peakall and Smouse, 2012). And the polymorphism information content index (PIC) was calculated by Popgene 1.32 software (Yeh et al., 1997). Based on the results of SSR marker detection, the amplification results were recorded using the “0-1” system. The bands with obvious peaks at the same position were marked as “1” and those without were marked as “0”, and the 0 and 1 matrices of SSR markers were established for each sample, and the matrix fingerprints of each sample were plotted.

## Results

### Identification and chromosome location of the *CCR* gene family

**Figure 1:**
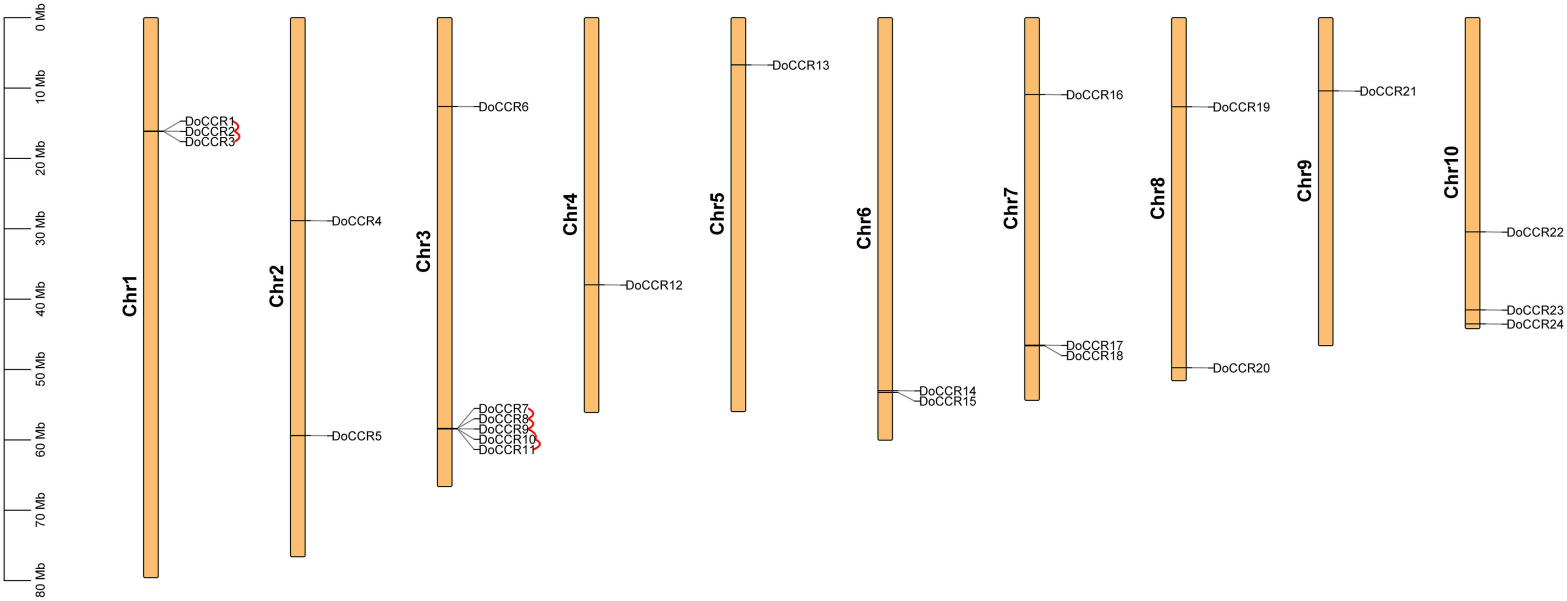
Chromosomal locations of *CCR* genes in *D. odorifera*. Each vertical bar indicates one chromosome. The chromosome number is shown at the left of each chromosome. Chromosomal locations of *DoCCRs* were mapped based on the *D. odorifera* genome. Red lines indicate tandem gene duplications.

A total of 24 full-length genes encoding putative CCRs were identified in the *D. odorifera* genome. These were named from *DoCCR*1 to *DoCCR*24 according to their position on the chromosome. The 24 *DoCCRs* were distributed on 10 chromosomes (**Figure 1**), among which chromosome 3 harbored the maximum number (6) of genes, while chromosome 1, 7 and 10 contained three genes each. Chromosomes 2, 6 and 8 contained two genes, while chromosome 4, 5 and 9 contained one gene each. In general, the central chromosome regions lacked *DoCCRs*, and half of genes were clustered on both the ends of chromosomes. This nonuniform distribution has also been found in *P. tomentosa* (Chao et al., 2017). These predicted 24 DoCCRs had peptide lengths (277-397 amino acids, aa) comparable to the known/functional CCRs, which were 319-379 aa (**Tables 1**; **S3**). Also, the other physicochemical properties of DoCCRs were similar to the validated CCRs from other plants (**Tables 1**; **S3**). Almost all functional CCRs were predicted to have an acidic pI with Ii values less than 40 and GRAVY values less than 0 (**Table S3**). Most of the CCR proteins (75%) were predicted and be acidic as their pI values ranged between 5.06 to 9.75 (**Table 1**). The Ii and GRAVY of the major CCR proteins were less than 40 and 0, respectively (**Table 1**). The subcellular localization predicted that the bulk of proteins was located in the endomembrane system, followed by the cytoplasm and other position (**Table 1**), while SiganlP results showed that all proteins had no signal peptide.

**Table 1:** Physicochemical properties and subcellular predictions of *CCR* gene family in *D. odorifera*.

Gene name	Gene ID	Length (aa)	MW (kDa)	pI	Ii	GRAVY	Subcell location
DoCCR1	evm.model.scaffold_273.160	326	36.09	6.20	30.53	-0.124	endomembrane system
DoCCR2	evm.model.scaffold_273.161_8	327	35.96	5.65	37.17	-0.026	endomembrane system
DoCCR3	evm.model.scaffold_273.161_1	327	36.01	6.27	32.08	-0.031	endomembrane system
DoCCR4	evm.model.scaffold_100.1815	319	35.28	5.69	34.48	-0.081	cytoplasm
DoCCR5	evm.model.scaffold_29.338	325	35.82	6.71	22.98	-0.089	endomembrane system
DoCCR6	evm.model.scaffold_206.822	389	43.11	6.65	44.55	-0.394	nucleus
DoCCR7	evm.model.scaffold_69.372_16	333	36.68	5.85	34.08	-0.005	endomembrane system
DoCCR8	evm.model.scaffold_69.372_15	325	35.92	6.03	39.37	-0.075	endomembrane system
DoCCR9	evm.model.scaffold_69.372_2	326	35.97	5.92	26.20	-0.044	cytoplasm
DoCCR10	evm.model.scaffold_69.372_1	325	35.45	6.54	27.06	0.034	endomembrane system
DoCCR11	evm.model.scaffold_69.373	318	34.72	9.75	33.07	-0.132	organelle membrane
DoCCR12	evm.model.scaffold_5.1342	339	36.79	6.04	29.59	0.015	endomembrane system
DoCCR13	evm.model.scaffold_35.575	313	33.98	5.06	27.75	-0.234	nucleus
DoCCR14	evm.model.scaffold_290.514	338	37.19	7.58	33.93	-0.172	cytoplasm
DoCCR15	evm.model.scaffold_290.537	322	35.61	8.39	35.61	-0.057	endomembrane system
DoCCR16	evm.model.scaffold_416.783	322	35.65	8.00	25.30	-0.118	endomembrane system
DoCCR17	evm.model.scaffold_457.457	331	36.71	5.34	36.95	-0.059	cytoplasm
DoCCR18	evm.model.scaffold_457.462	357	39.20	8.54	30.41	-0.222	endomembrane system
DoCCR19	Novelgene0905	320	35.08	5.79	30.44	0.072	endomembrane system
DoCCR20	evm.model.scaffold_162.78	397	44.72	8.76	38.75	-0.202	chloroplast
DoCCR21	evm.model.scaffold_42.76	330	36.46	5.24	34.54	-0.137	endomembrane system
DoCCR22	evm.model.scaffold_40.396	359	39.32	5.61	36.59	0.027	plasma membrane
DoCCR23	evm.model.scaffold_40.1434	331	36.91	6.36	42.58	-0.125	organelle membrane
DoCCR24	evm.model.scaffold_40.1658	277	29.88	6.14	33.26	0.005	organelle membrane

MW, molecular weight; pI, isoelectric point; Ii, instability index; GRAVY, grand average of hydropathicity.

### Conserved domain and gene structure analysis

**Figure 2:**
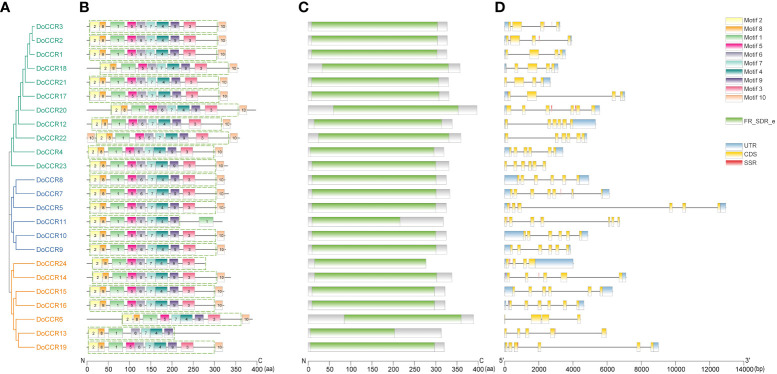
Phylogenetic relationship, conserved motifs, conserved domains and gene structures of the DoCCR family. **(A)** The phylogenetic tree of all DoCCR proteins was constructed using neighbor-joining (NJ) method with 1,000 bootstrap. Different branch colors represent different groupings. **(B)** Conserved motifs in DoCCR proteins. The motifs, numbers 1-10, were displayed in different colored boxes. The green dashed box indicates the FR_SDR_e domain corresponding to the one in **(D)**. **(C)** The conserved domain in the DoCCR proteins. Green boxes indicate FR_SDR_e domain, which identified by CDD database. The units on the bottom scale are the number of amino acids. **(D)** The exon-intron structure of *DoCCR* genes. Exons and introns were indicated by boxes and single lines. The thin blue boxes represent UTRs, yellow boxes represent CDSs and grey lines represent introns. Additionally, the red boxes represent SSR (excluding mononucleotide repeats).

**Figure 3:**
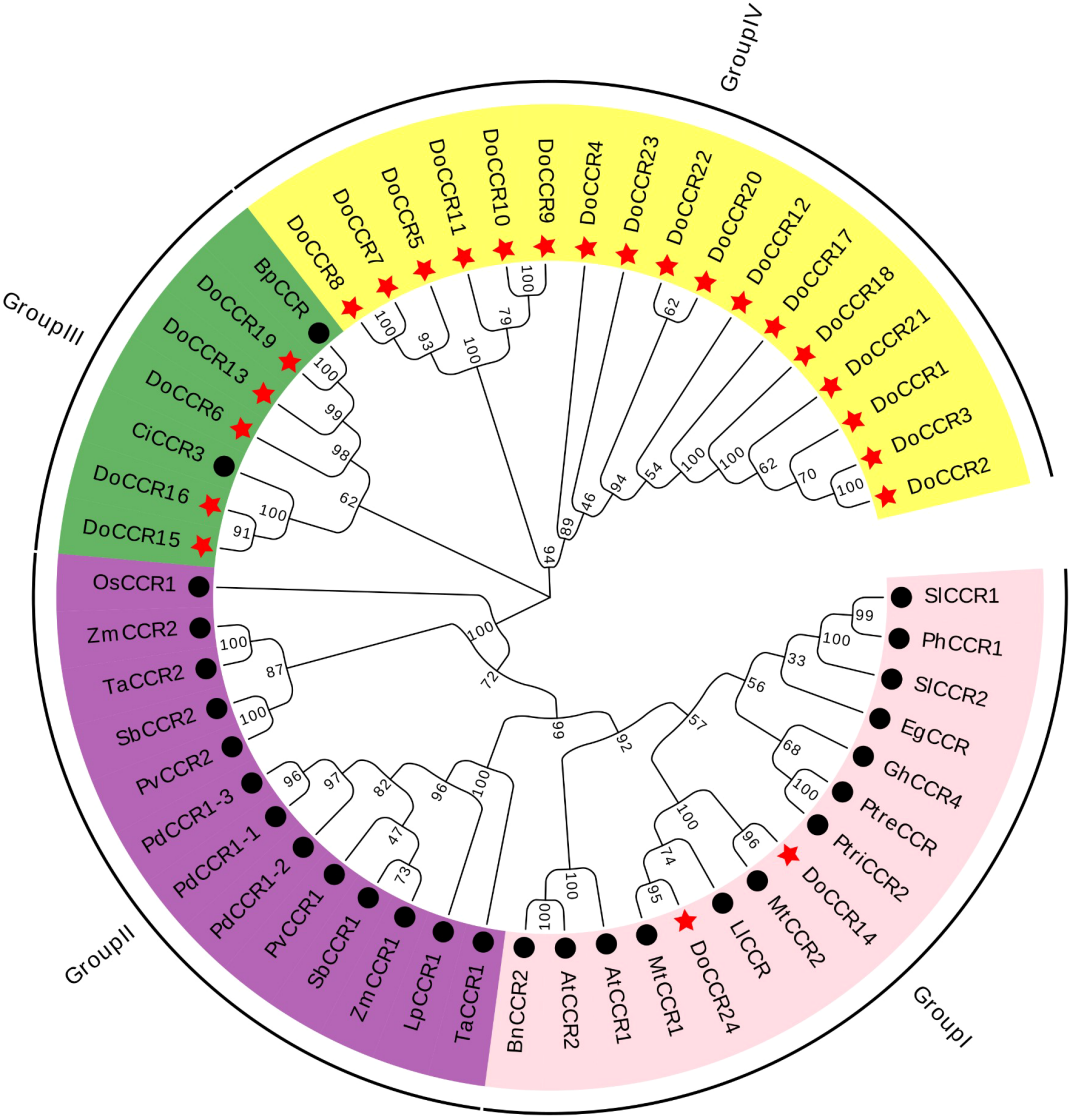
Phylogenetic tree of DoCCRs and 28 charaterized CCRs from other plant species. The neighbor-joining (NJ) tree was constructed by MEGA11 with 1,000 bootstrap replicates. The purple shade indicates monocot CCRs, and other shades indicate dicot CCRs. The red star indicates the DoCCRs and the black circle indicates the functional CCRs from other plants. At, *A*. *thaliana*; Bp, *B*. *platyphylla*; Bn, *B. napus*; Ci, *C. intermedia*; Do, *D*. *odorifera*; Eg, *E. gunnii*; Gm, *G. mexicanum*; Ll, *L. leucocephala*; Lp, *L. perenne*; Mt, *M. truncatula*; Os, *O. sativa*; Pv, *P. virgatum*; Pd, *P. dilatatum*; Ph, *P. hybrida*; Ptre, *P. tremuloides*; Ptri, *P. trichocarpa*; Sl, *S. lycopersicum*; Sb, *S. bicolor*; Ta, *T. aestivum*; Zm, *Z. mays*. Detailed information of all CCRs from other plants are showed in **Table S1**.

Further analysis of the gene structure, conserved motifs and structural domains in the DoCCR family was shown in **Figure 2**. First, phylogenetic analysis of the 24 DoCCR proteins divided them into three subfamilies (**Figure 2A**), among which the topological structure was similar to that of a phylogenetic tree constructed using CCRs from 19 plant species (**Figure 3**). To investigate the diversity in the gene structures among the 24 *DoCCR*s, their exon-intron arrangement was visualized, as shown in **Figure 2D**. From the phylogenetic clusters, we observed that the members in the same clade displayed similar structures. The number of exons in the 24 *DoCCRs* was ranged from 4 to 7, with *DoCCR7* and *DoCCR11* having 7 exons, *DoCCR13* and *DoCCR14* having 5 exons, whereas the remaining genes contained 4 or 6 exons. A total of 10 conserved motifs in DoCCR proteins (motif 1 to motif 10) were found using MEME tool. Most DoCCRs contained these 10 motifs, but some (DoCCR6, DoCCR11, DoCCR13, DoCCR22 and DoCCR24) had individual motif deletions or duplications (**Figure 2B**). Irrespective of their phylogenetic bearing, or gene structures, most of the motifs were conserved across DoCCRs (**Figure 2**). Meanwhile, Each DoCCR protein had a conserved domain FR_SDR_e (flavonoid reductase (FR), extended (e) SDR; cd08958), which was identified by CDD database to obtain. Most of the domains were composed of motif 1 to motif 9 in the order shown in the **Figure 2B**, but the domains of DoCCR11 and DoCCR13 did not contain motif 3. Additionally, the domains of DoCCR11 and DoCCR13 were shorter compared to those of other DoCCRs. Furthermore, earlier research revealed that expanded SDRs contained a about 100 amino acid C-terminal extension that was less conserved (Kavanagh et al., 2008). It indicated that motif 3 may be an optional component of the domain.

### Sequence homology and phylogenetic analysis

The primary structures of most known CCR enzymes, including a characteristic NAD(P) binding Rossmann fold and a conserved Ser-Tyr-Lys catalytic triad, were present in all of the identified DoCCRs (**Figure S1**) (Kallberg et al., 2002; Chao et al., 2017). The existence of the conserved motifs involved in NADP(H) binding and substrate catalysis was discovered by multiple alignment of these DoCCRs together with functional CCRs from other species. Motif G-X-X-G-X-X-A and D-X-X-D were relatively conserved in most DoCCRs, and they were engaged in NAD(P) binding and adenine binding pocket stability (Chao et al., 2017). Additionally, the NADP specificity motif R(X)_5_K was discovered, which is a crucial characteristic that sets the CCR apart from other NAD(H)-dependent SDRs (Pan et al., 2014). However, DoCCR6, DoCCR13 and DoCCR19 did not contain this motif, while R(X)_3_K, R(X)_6_K and R(X)_7_K were found in a few DoCCRs (**Figure S1**), which may affect the utilization of NADPH (Chao et al., 2017). Only DoCCR6 lacked the crucial catalytic site S220, which is part of a catalytic triad with Y260, K264 (**Figure S1**). The reported CCR signature motif NWYCYGK may be essential for CCR activity (Lacombe et al., 1997; Park et al., 2017), where the Y(X)_3_K catalytic center can be found in all DoCCRs (**Figure S1**). A few substitutions were found on the second aa (W), which was similar to the CCRs in poplar (Chao et al., 2017). In particularly, DoCCR14 and DoCCR24 had complete motif NWYCYGK, and DoCCR15 and DoCCR16 had only one substitution on the sixth aa (S replaced G).

To study the evolutionary relationship between *DoCCRs and* functional *CCR* genes from other plant species, a total of 28 CCR proteins were retrieved from *A. thaliana*, *B. napus*, *B. platyphylla*, *C. intermedia*, *E. gunnii*, *G. mexicanum*, *L. leucocephala*, *L. perenne*, *M. truncatula*, *O. sativa*, *P. dilatatum*, *P. hybrida*, *P. tremuloides*, *P. trichocarpa*, *P. virgatum*, *S. lycopersicum*, *S. bicolor*, *T. aestivum*, and *Z. mays* (**Table S1**). Multiple alignment of CCR protein sequences revealed that DoCCR1-24 had about 38-90% similarity to functional CCRs from other plant species. In particularly, DoCCR5, DoCCR7-10, DoCCR14-16, DoCCR19 and DoCCR24 were highly homologous (63-90% similarity) with known CCRs (**Table S4**). It was surprising that DoCCR13 had high similarity, about 61%, with BpCCR, but low similarity with other known CCRs (less than 50%). The phylogenetic analysis using MUSCLE and MEGA11 divided CCR proteins into four groups (**Figure 3**). Among 24 DoCCRs, DoCCR14 and DoCCR24 together with MtCCR1, MtCCR2, LlCCR and other dicotyledons were grouped in Group I, while DoCCR15, DoCCR16 and CiCCR3 were grouped in Group III, and DoCCR6, DoCCR13, DoCCR19 together with BpCCR were also grouped in Group III. The remaining DoCCRs were distributed to group IV. All functionally characterized CCRs from monocots were clustered in Group II, which did not contain any DoCCRs.

### Gene duplication and synteny analysis

The BLASTP and MCScanX programs were used in the present study to reveal the origin of gene duplication for the *CCR* gene family in *D. odorifera*. Two tandem duplication gene pairs were observed on chromosome 1 and four tandem duplication gene pairs were located on chromosome 6, and these homologous genes formed two gene clusters on the corresponding chromosomes, respectively (**Figure 1**). In addition, three segmental duplication gene pairs were detected between different chromosomes (1, 4, 6, 7, 10) of the *D. odorifera* genome, respectively *DoCCR1* & *DoCCR17*, *DoCCR12* & *DoCCR22*, *DoCCR14* & *DoCCR24* (**Figure S2**). A total of 12 genes were implicated in gene duplication events, and these findings imply that *DoCCRs* may be generated by tandem duplication or segment duplication, and that these duplication events were the primary causes of the *CCR* gene family’s expansion in *D. odorifera*. Furthermore, Ka/Ks ratios of these duplicated genes were calculated to identify the driving factor behind the development of the *DoCCR* gene family. The Ka/Ks ratios of the nine gene pairs that experienced tandem or segment duplication events were all smaller than 1, ranging from 0.15 to 0.57 (**Table S5**), suggesting that purifying selection had a major role in the development of *CCR* genes in *D. odorifera*.

**Figure 4:**
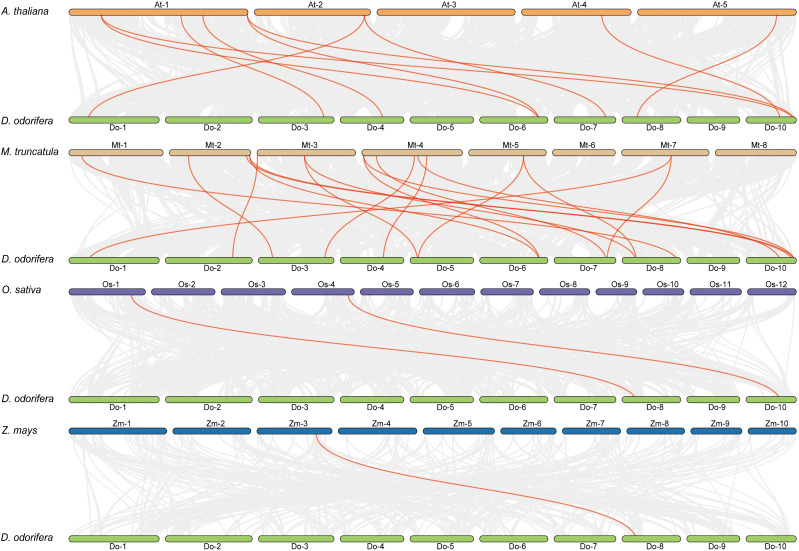
Syntenic relationships of *DoCCR* genes with other CCR genes in four representative plant species. Gray lines in the background indicated the collinear blocks within *D. odorifera* and other plant genomes. Red lines in highlight indicated the syntenic CCR gene pairs.

Previous research has demonstrated that *D. odorifera* underwent a fresh round of whole-genome duplication (WGD) following the alleged ancient γ WGD event shared by core eudicots (Hong et al., 2020). To further uncover the evolutionary mechanisms of the *DoCCR* gene family, we conducted a synthetic analysis of *CCR* genes between *D. odorifera* and four representative species, including two dicots (*A. thaliana* and *M. truncatula*) and two monocots (*O. sativa* and *Z. mays*) (**Figure 4**). The numbers of orthologous gene pairs between *D. odorifera* and the other four species (*M. truncatula*, *A. thaliana*, *O. sativa* and *Z. mays*) were 18, 10, 2 and 1, respectively (**Table S6**). And there were 14, 8, 2 and 1 *DoCCRs* showing colinear relationships with *CCRs* in *M. truncatula*, *A. thaliana*, *O. sativa* and *Z. mays*, respectively. Different numbers of orthologous gene pairs between *D. odorifera* and other species were correlated with divergence in evolutionary time (**Figure 4**). In general, *D. odorifera* had far more orthologous gene pairs with dicotyledons than with monocotyledons. Among dicots, *D. odorifera* and *M. truncatula* had more orthologous gene pairs than *D. odorifera* and *A. thaliana*. This is in line with the fact that *D. odorifera* and *M. truncatula* are more closely related phylogenetically than A. thaliana (Hong et al., 2020). Furthermore, six collinear gene pairs (*DoCCR1*, *DoCCR7*, *DoCCR12*, *DoCCR14*, *DoCCR17*, *DoCCR24*) were found in *D. odorifera* and *M. truncatula*/*A. thaliana* but not in *D. odorifera* and *O. sativa*/*Z. mays*, which indicated that these orthologous gene pairs may have developed after the divergence of dicotyledons and monocotyledons. Six collinear gene pairs (*DoCCR5*, *DoCCR6*, *DoCCR13*, *DoCCR18*, *DoCCR20*, *DoCCR23*) in *M. truncatula* were not present in *A. thaliana*, implying that these orthologous pairs may have originated in the ancestor of the legume family. Additionally, it was discovered that *D. odorifera* and the other four species shared a collinear pair, suggesting that the orthologous pairs may have existed prior to the ancestral divergence.

### Promoter cis−acting elements analysis

**Figure 5:**
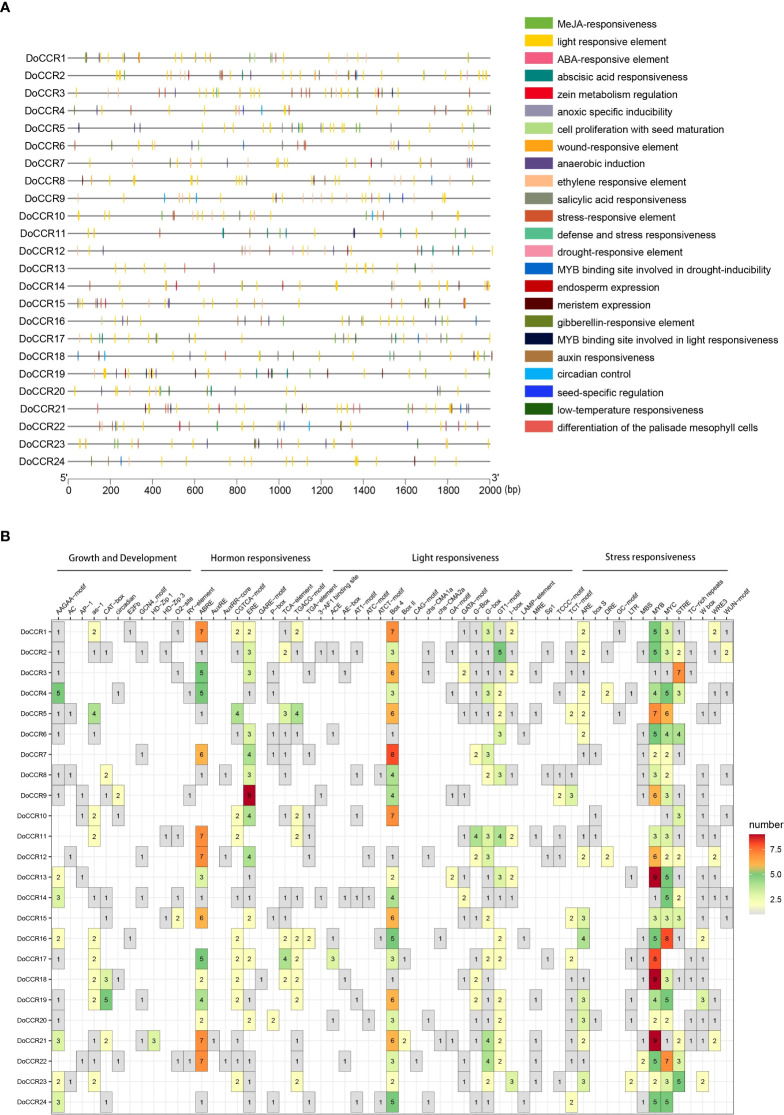
Representation of cis-acting elements in the promoter regions of *DoCCR* genes. **(A)** Distribution of different kinds of cis-elements in the upstream 2,000 bp sequences of the *DoCCRs*. Different color boxes correspond to the identity of cis-elements; some cis-elements may overlap with others. **(B)** The number of cis-elements in the promoters of the *DoCCRs*.

To further understand gene function and regulation patterns, cis-acting elements in the *DoCCR* gene promoter sequences were investigated. A region 2,000 bp upstream of the start codon of each *DoCCR* gene was determined using PlantCARE. These cis-elements were divided into four categories, namely stress responses, light responses, hormone responses and growth and development related elements (**Figure 5**). Although the cis-element distributions in these genes varied, several common characteristics could be seen. Compared with the other three types, the number of cis-elements for growth and development was relatively small, but the promoters of each gene contained this type of elements. Of these, AAGAA-motif, which is involved in secondary xylem development (Hou et al., 2021), was the most abundant and could be detected in 17 promoters. Stress and hormone response elements were abundant in the promoters, particularly MYB, MYC and STRE (stress response elements), ABRE (abscisic acid response element), ERE (ethylene response element) and CGTCA motifs (methyl jasmonate response element). Other cis-elements related to stress responses were also identified, such as ARE, DRE, LTR, WUN-motif, which represented plant responses to anaerobic induction, drought, low temperature and wounding stresses. TGACG-motif was found in some promoters lacking CGTCA-motif, which could also be involved in the methyl jasmonate response. MYB-related elements were detected in 23 promoters (excluding *DoCCR10*), while MYC-related elements were found in the remaining promoters except *DoCCR17*. Additionally, a large number of widely distributed light responsive elements were observed, including Box 4, G-box and GT1-motif, with Box 4 being the most numerous and present in 23 promoters. G-box may interact with the transcription factors bZIP or bHLH (Ezer et al., 2017; Liu et al., 2022). The abundance and diversity of cis-elements provide additional evidence for the broad roles of *DoCCRs*.

### Protein-protein interaction network of DoCCR proteins

To investigate the relationship between *DoCCRs* and other genes, a protein-protein interaction network was constructed using the STRING database. Four *DoCCRs* were predicted to interact with 10 different proteins (**Figure S4**). These interaction proteins were mainly involved in flavonoid synthesis (F3H, FLS1, FLS3, TT5, and TT7) or lignin synthesis (CYP84A1). The phenylpropane pathway included C4H and HCT, which were crucial in supplying precursors for the secondary metabolism of xylitol and flavonoids. Additionally, DoCCR20 had the most interaction partners, while DoCCR14 and DoCCR24 could interact with each other. These results will be beneficial to future research and verify *DoCCRs* biological function based on relevant experiments.

### Expression patterns of *DoCCR* genes based on RNA-seq data

**Figure 6:**
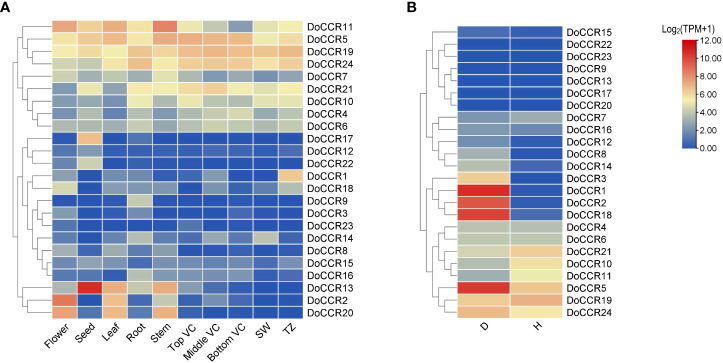
Expression profiles of *DoCCR* genes in different tissues and under abiotic stress. **(A)** The accumulation patterns of *DoCCRs* in different tissues, such as flower, seed, leaf, root, stem, top (Top VC), middle (Middle VC), and bottom (Bottom VC) of vascular cambium, sapwood (SW), transition zone (TZ). **(B)** The expression patterns of *DoCCRs* in stems after wounding treatment. Discolored (D), and healthy (H) zones of pruning-induced stems were assayed. Scale bars on the right represent log_2_(TPM+1) transformed values.

To determine the physiological roles of *CCR* genes in *D. odorifera*, we further examined the transcriptome data of *DoCCRs* in multiple tissues and under wounding stress (**Figure 6**). The expression profiles indicated that a small number of *DoCCRs* were expressed in all tissues, while nearly half of the genes were not expressed in one or more tissues (**Figure 6A**). The *DoCCR11* was highly expressed in flower, leaf and stem tissues, exhibiting weak expression in Bottom VC. Although *DoCCR5*, *DoCCR19* and *DoCCR24* were expressed in both vegetative and reproductive organs, they were all highly expressed in different parts of the vascular cambium. Meanwhile, *DoCCR5* was also highly expressed in stems, and *DoCCR19* and *DoCCR24* were also highly expressed in roots and xylem (SW and TZ). Some genes displayed tissue-specific expression, including *DoCCR*1 accumulated only in TZ, *DoCCR*3 in flowers, *DoCCR*9 and *DoCCR*16 in roots, *DoCCR*12, *DoCCR*17 and *DoCCR*22 in seeds. *DoCCR*23 was only expressed in flowers and Top VC but not in other tissues. *DoCCR*14 was highly expressed in roots and SW, whereas *DoCCR*18 in flowers and TZ.

Lignification is among the most crucial defenses against stressors. The involvement of *CCRs* in regulating wounding stresses has been confirmed in various plants (Ghosh et al., 2014). Thus, fluctuations in the transcripts of *DoCCR* genes in response to wounding were studied based on RNA-seq data. The results indicated that nine *DoCCRs* (*DoCCR*1, *DoCCR*2, *DoCCR*5, *DoCCR*8, *DoCCR*12, *DoCCR*14, *DoCCR*15, *DoCCR*18, *DoCCR*24) were upregulated (TPM of D zone/TPM of H zone ≥ 2) in D zone compared with H zone. Of these, seven genes (*DoCCR*1, *DoCCR*2, *DoCCR*5, *DoCCR*8, *DoCCR*12, *DoCCR*14, *DoCCR*18) were upregulated by ≥ 15 fold (**Figure 6B**). Furthermore, of the seven genes, only *DoCCR5* had a TPM ≥ 1 in H zone. In particularly, *DoCCR*3 was expressed in D zone and not expressed in H zone. It was surprise that five genes (*DoCCR*7, *DoCCR*10, *DoCCR*11, *DoCCR*19, *DoCCR*21) show downregulated after three weeks of pruning. Six genes (*DoCCR*9, *DoCCR*13, *DoCCR*17, *DoCCR*20, *DoCCR*22, *DoCCR*23) were not detected in both D and H zones (**Figure 6B**), while these genes were not expressed in xylem, middle VC and bottom VC (**Figure 6A**).

### SSR mining, characteristic and distribution

The 24 *DoCCR* gene sequences were scanned with the help of MISA software. A total of 74 SSR loci were obtained, where 19 genes contained two or more SSRs, with an average of one SSR per 1.70 kb, with an incidence of 91.67% (**Tables 2**; **S7**). Among the 24 *DoCCR* genes, all SSR loci were distributed in the non-coding region, mainly in the intron region (67, 90.54%), and a few in the UTR, including 5’UTR (5, 6.76%) and 3’UTR (2, 2.70%) (**Figure 2**; **Table S7**). The mononucleotide repeats accounted for the majority of SSRs (52, 70.27%), followed by di- (13, 17.57%), tri- (8, 10.81%) and penta-nucleotide (1, 1.35%) repeats (**Table S8**). These repeat motifs were further characterized by SSR length and repeat number. The number of SSR repeat length ranged from 10 to 40 bp with 10, 11 and 12 bp repeat length the most recurrent. Among mono-, di-, tri-, and penta-nucleotide repeats, 10/11 (26.92%), 5/6 (33.33%), 5 (100%) repeat number were most common (**Table S7**). The mono-repeat A/T was the most abundant motif detected in all SSRs (48, 64.86%), followed by AT/TA (8, 10.81%), AAG/CTT (4, 5.41%), and C/G (4, 5.41%), among others (**Table S8**).

**Table 2:** The SSR loci in *DoCCR* genes (excluding mononucleotide repeats).

Gene Name	SSR	Start	End	Length	Location
DoCCR2	(TA)11	316	337	22	Intron
DoCCR2	(TA)17	2011	2044	34	Intron
DoCCR3	(TA)7	2324	2337	14	Intron
DoCCR3	(TA)6	2766	2777	12	Intron
DoCCR5	(TTTTA)5	303	327	25	Intron
DoCCR5	(TAT)7	757	777	21	Intron
DoCCR7	(TTA)7	3277	3297	21	Intron
DoCCR9	(TTC)5	2758	2772	15	Intron
DoCCR11	(TGT)6	171	188	18	Intron
DoCCR11	(TG)6	2107	2118	12	Intron
DoCCR13	(ATA)5	2972	2986	15	Intron
DoCCR14	(TC)12	46	69	24	5’UTR
DoCCR14	(TA)9	2003	2020	18	Intron
DoCCR16	(GAA)6	252	269	18	5’UTR
DoCCR17	(AT)6	164	175	12	5’UTR
DoCCR19	(TTC)5	233	247	15	Intron
DoCCR19	(TC)7	762	775	14	Intron
DoCCR19	(TCT)6	773	791	18	Intron
DoCCR20	(AT)20	2731	2770	40	Intron
DoCCR20	(TA)19	4130	4167	38	Intron
DoCCR23	(AC)6	1198	1209	12	Intron
DoCCR24	(TC)17	286	319	34	Intron

### SSR primer design, screening and polymorphism analysis

Primer pairs were designed for 22 SSR loci (remove mononucleotide repeats) in 14 *DoCCR* genes using Primer3 software, and after a series of screening 9 primer pairs (one SSR loci on each gene) were finally selected for synthesis. They were used to PCR-amplify a set of 10 *D. odorifera* samples from geographically distant templates, and products were detected by capillary electrophoresis. Only one pair of primers (CCRS2) produced clear and reproducible amplicons of the expected size, and it could amplify more than 2 specific bands (**Figure S3**; **Table S9**).

**Figure 7:**
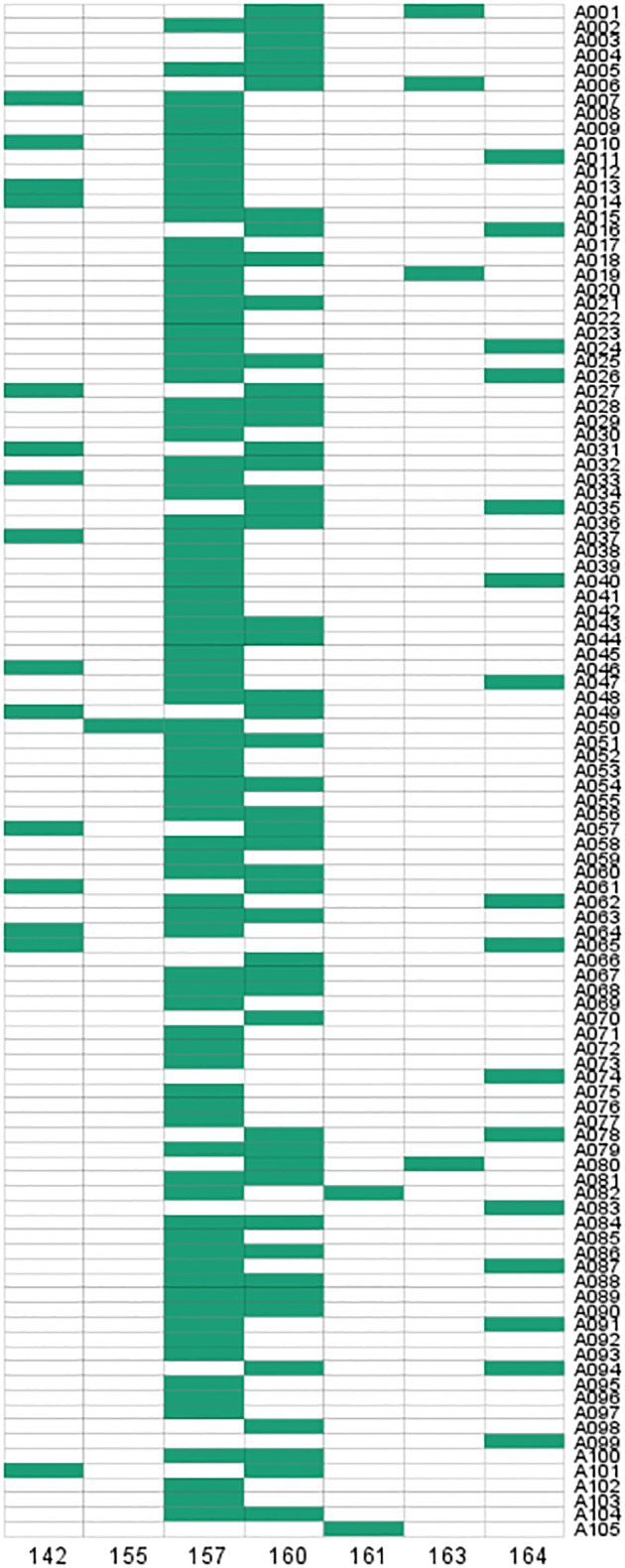
Fingerprint analysis of 105 *D. odorifera* samples from SSR loci in *DoCCCR5*.

To verify the validity of the SSR primers, PCR amplification was performed in 105 *D. odorifera* individuals. The results showed that 7 alleles were observed in 105 samples and specific bands could be amplified in each sample (**Figure 7**; **Table S10**). The effective number of alleles (*Ne*) for this locus was 2.669, the observed heterozygosity (*Ho*) was 0.610, the expected heterozygosity (*He*) was 0.625, and the fixation Index (*F*) was 0.025 (**Table S10**). The Shannon diversity index (*I*) was 1.248 (**Table S10**), indicating high genetic polymorphism. The polymorphism information content (PIC) was 0.586 (**Table S10**), which was higher than 0.5, indicating that the genetic marker provided a high degree of information that can be used to further investigate the genetic diversity of the natural population. In addition, this SSR marker can also amplify specific products in other *Dalbergia* plants (*D. tonkinensis*, *D. sissoo*, *D. cochinchinensi*) and *P. santalinus*, indicating that it has good cross-species transferability.

### Genetic diversity of the main species sources of *D. odorifera*

Genetic diversity analysis was performed on 85 individuals from seven geographical populations of *D. odorifera* (discarding some populations that were too small to analyze) (**Table S11**). The range of variations in *Na* among the different populations was from 3.000 (LG) to 5.000 (HK), and the mean value was 4.143. For *Ne*, the range was from 2.000 (LG) to 2.810 (HK), with an average of 2.480. For *I*, variations ranged from 0.868 (LG) to 1.255 (HK), with a mean value of 1.098. Variations in *Ho* ranged from 0.500 (LG) to 0.833 (DF), with a mean of 0.656, whereas variations in *He* ranged from 0.500 (LG) to 0.644 (HK), with a mean value of 0.592. The LG population had an *F* of 0, showing that its genotype frequencies were consistent with Hardy-Weinberg theoretical ratios, while the other six populations had *F* values less than 0, indicating that there were more heterozygous individuals than pure individuals in these groups. Furthermore, the HK population showed high genetic diversity (*Na* = 5.000, *He* = 0.644, and *I* = 1.255), whereas the LG population exhibited a relatively low level of genetic diversity (*Na* = 3.000, *He* = 0.500, and *I* = 1.868).

## Discussion

### Identification of *CCR* gene family in *D. odorifera*

The *CCR* gene family is widely distributed in plants, and these *CCR* or *CCR-like* genes have been identified in gymnosperms, angiosperms, ferns, and even in some photosynthetic algae (Barakat et al., 2011). Despite being a sizable gene family, only a tiny subset of *CCR* genes have been found to encode biochemically functional CCRs for the lignin and defense-related phenolic compound biosynthesis (Barakat et al., 2011; Park et al., 2017). In order to identify the bona fide *CCR* genes, we used two methods to comprehensively collect all possible CCRs from the genome of *D. odorifera*. After verification of domains in multiple databases, we retained the sequences that were consistent with those experimentally verified CCRs in other species. These DoCCRs have similar physicochemical properties to the functionally validated CCRs, except that two of them were surprisingly long, especially as some of their introns were much larger than the previously studied CCRs. These two genes also contained multiple domains, whereas functionally characterized CCRs had only one domain in other plants. Therefore, we used FGENESH program (Solovyev et al., 2006) to re-perform gene annotation based on legumes and model plant parameters models and combined with the long-read PacBio sequences obtained from the previous study to assist annotation (Zhao et al., 2022). Finally, we predicted six new genes that passed all the domain verifications and had similar physicochemical properties as the previously characterized CCRs.

These 24 DoCCR proteins show high homology to well conserved NAD(P)-binding domain in the N-terminal region, which is a common feature of the NAD(P)H-dependent SDR family (Chao et al., 2017). The NADP-specificity motif, R(X)_5_K, is a key structural feature that distinguishes CCRs from the NAD(H)-specific SDRs (Pan et al., 2014). Three variants of this motif, R(X)_3_K, R(X)_6_K and R(X)_7_K, were frequently found in plants (Chao et al., 2017). But DoCCR6, DoCCR13 and DoCCR19 did not contain R(X)_5_K or other variants, which may greatly reduce their activity. CCR have a signature motif NWYCYGK, which is thought to be a feature of the catalytic site (Lacombe et al., 1997; Chao et al., 2017). Among 24 DoCCRs, DoCCR14 and DoCCR24 exhibited the fully conserved catalytic motif, whereas DoCCR15 and DoCCR16 had the signature motif with two amino acid variation, which was consistent with the CiCCR3. The G to A substitution in the CCR catalytic motifs has been frequently found in other active CCRs, such as in PvCCR2 and ZmCCR2 (**Figure S1**) (Park et al., 2017). Most of the DoCCRs except DoCCR17 and DoCCR21 were very conserved in the WYK amino acids, and similar situations have been reported in poplar (Chao et al., 2017). The catalytic triad, Ser–Tyr–Lys, in the SDR family members have been shown to be critical for catalysis (Filling et al., 2002; Kallberg et al., 2002). It is possible that DoCCR6 might have lost its CCR activity, because it might not use NADPH due to its loss of serine residue in the catalytic triad. H(X)_2_K is an essential substrate binding motif (CCR-SBM) and ensures the CCR activity, which could be used as additional criteria to distinguish CCRs from CCR-like proteins (Chao et al., 2019). The existence of H(X)_2_K in DoCCR was similar to the patterns observed for the NWYCYGK motif: only DoCCR14 and DoCCR24 contained this structure, while V(X)_2_K was found in DoCCR15 and DoCCR16, consistent with CiCCR3. Variations in H(X)_2_K have also been observed: The K in OsCCR1 was substituted by R, whereas BpCCR did not contain this motif. OsCCR1, with four mismatches in the NWYCYGK motif (NLYCCAK), displayed no observable activity toward the hydroxycinnamoyl-CoA substrate under normal circumstances (Park et al., 2017), while OsCCR1 was substantially activated by coincubation with the small GTPase OsRac1 that regulated defense-related lignin synthesis (Kawasaki et al., 2006). It implied that some DoCCRs might require additional cofactors to stimulate their activity. CCR showed substrate adaptation to a variety of hydroxycinnamoyl CoA, but exhibited different affinities for different substrates. Different CCRs differed in their substrate catalytic efficiency and preference. For example, PvCCR1 and MtCCR1 showed substrate preference for feruloyl CoA, whereas PvCCR2 and MtCCR2 showed preference for caffeoyl CoA and p-coumaroyl CoA (Escamilla-Treviño et al., 2010; Zhou et al., 2010). The divergence of the CCR in substrate adaptation may be responsible for the variation in DoCCR catalytic motifs, which needs to be further systematically investigated on enzyme activities based on different substrates.

### *DoCCR* genes involved in growth and stress response

The cis-acting elements in the promoter region are very important for gene expression. The analysis of promoters of *DoCCRs* indicated the presence of abundant light-, hormone-, stress-response and developmental elements. These promoters contained many light response elements, such as Box4 and G-box. Light signaling directly impacts the transcription of structural genes and transcription factors involved in lignin metabolism, which in turn affects lignin monomer production and cell growth (Zoratti et al., 2014). For example, light can alter sugar metabolism in fruit, and sugars not only serve as precursors and energy for lignin metabolism, but also act as signaling molecules to indirectly controlling lignin synthesis (Roy, 2016; Cheng et al., 2017). Additionally, the majority promoters had cis-elements related to the abscisic acid, ethylene, and methyl jasmonate response, suggesting that these genes are crucial in the response to a variety of biotic and abiotic stressors. All promoters contained MYB or MYC transcription factor recognition or binding sites. One of the most significant groups of transcription factors in plants, MYB and MYC are involved in the control of several stress-resistance mechanisms, plant growth and development, the manufacture of secondary metabolites, and signal transmission involving plant hormones (Ren et al., 2022). It was discovered that the pine and *Eucalyptus* MYB46 homologs (PtMYB4 and EgMYB2) function in the control of lignin synthesis (Patzlaff et al., 2003; Goicoechea et al., 2005). In addition to controlling the expression of the genes involved in monolignol biosynthesis, a variety of MYB and other transcription factors also bind to AC elements, transactivating the corresponding promoters (Raes et al., 2003). In *Arabidopsis*, MYB46 and MYB83 could activate the expression of *AtCCR1* by binding to AC elements in their promoters (Xie et al., 2018). *DoCCR5*, *DoCCR8* and *DoCCR12* were detected to contain AC elements, which may have a similar mechanism of action to MYB or other transcription factors. The majority of *DoCCRs* had AAGAA-motifs in their promoters that are related to the development of secondary xylem (Hou et al., 2021), indicating that these genes may regulate the accumulation of lignin in the xylem secondary cell wall during development.

Phylogenetic analysis demonstrated that CCRs were classified into four groups, where the genes of monocotyledons form a completely separate cluster (Group II). We found that seven DoCCR proteins (DoCCR6, DoCCR13, DoCCR14, DoCCR15, DoCCR16, DoCCR19, DoCCR24) have a close evolutionary relationship with the functional CCR from dicotyledons. In Group I, DoCCR14 and DoCCR24 were clustered with MtCCR1, MtCCR2, and LlCCR to form an evolutionary branch. *M. truncatula* harboring transposon insertions in *MtCCR1* exhibited drastically reduced growth and lignin content, whereas *MtCCR2* knockouts grew normally with moderate reduction in lignin levels (Zhou et al., 2010). CCR’s role in lignification in *L. leucocephala* was successfully by over-expressing and knocking down *LlCCR* expression with the help of sense and antisense constructs (Prashant et al., 2011). *DoCCR14* was highly expressed in actively lignifying tissues, including xylem and roots. In the two regions of xylem, *DoCCR14* was only highly expressed in SW, which may indicate that *DoCCR14* plays an important role in the heart wood formation while participating in lignin synthesis. *DoCCR24* was expressed in all the tissues, and was highly expressed in xylem, roots and vascular cambium, which indicates that its function might have additional functions, not limited to lignifying areas. Interestingly, we found DoCCR6, DoCCR13, DoCCR15, DoCCR16 and DoCCR19 grouped together in Group III along with previously characterized CiCCR3 and BpCCR, which were associated with lignin synthesis. *DoCCR13* and *DoCCR16* displayed tissue-specific expressions in seeds and roots, respectively. *DoCCR6* and *DoCCR24* have similar expression patterns, which were highly expressed in roots, vascular cambium and xylem. *DoCCR15* has low expression in all the tissues, whereas *DoCCR19* was highly expressed in the xylem and vascular cambium. 17 DoCCR proteins were clustered into Group IV, which was far away distant from the known genes from other species, indicating that they may be *CCR*-like genes or the unique *CCRs* of *D. odorifera*. Interestingly, *DoCCR9* was specifically expressed in roots while *DoCCR23* was only slightly expressed in flower and top VC, but they both hardly expressed in other tissues or D/H zone. Nearly half of the genes were induced to be expressed under wound treatment and some of them, such as *DoCCR1*, *DoCCR5* and *DoCCR18*, were strongly induced to be up-regulated in expression, which may indicate their function in relation to the response to wounding stress.

### SSR prediction, validation, and application

Compared with other markers, such as random amplified polymorphic DNA (RAPD) and sequence-related amplified polymorphism (SRAP), SSR markers have the advantages of high polymorphism, co-dominance, less DNA consumption, good test repeatability and reliable results (Taheri et al., 2018). They have been widely used in genetic research of forest tree species and molecular marker assisted early selection breeding. Therefore, the development of species-specific SSR markers is highly required for the research related to molecular genetics and functional genomics, such as the evaluation of genetic diversity of important tree species, the mapping/fingerprinting, germplasm identification, the gene localization and the molecular marker assisted selection. Although some SSR markers were previously developed by using the leaf transcriptome (Liu et al., 2019b), there may be two problems due to the characteristics of RNA itself. First, due to the spatiotemporal specificity of the transcriptome, the expression of some genes in selected tissues or organs is too low or even not expressed to be detected; Second, the gene structure represented by the transcript is not complete, and the information of introns and upstream and downstream regulatory regions is missing, which leads to the poor detection of SSR loci in these regions. However, functional genes are generally subject to strong natural selection in the process of evolution. As SSR loci within genes are also subject to strong selection pressure like the genes themselves, the result is that polymorphic SSR loci within genes are often closely linked to the formation of phenotypes or traits of a species (Li et al., 2004). If SSRs occur in the coding region of a gene, it may affect protein quality, resulting in a difference in quality; On the other hand, if it located in the regulatory or intronic regions, it may affect the gene expression and RNA stability (Li et al., 2004). SSRs located in UTR may affect gene expression and phenotype, in which SSR amplification in 3’UTR could lead to transcriptional slippage, while intronic SSRs affect gene transcription, correct splicing of mRNA, or export to the cytoplasm (Varshney et al., 2005; Vieira et al., 2016). 74 SSRs identified in 24 *DoCCRs* show a wide-spread occurrence in UTR and intronic regions, and might have profound implications for the expression and function of these genes.

The usefulness and success of employing SSRs heavily depend on the markers’ quality, the precision of the genotyping data, and the chosen plant materials (Liu et al., 2017). Thus, the validation of the identified SSRs is an important part of the process of establishing a workable marker sets for genetic improvement work. Of these 9 primer pairs, only one (CCRS2) in *DoCCR5* yielded clear bands across the 10 *D. odorifera* trees. Therefore, it was finally selected to amplify and verified in 105 samples obtained from natural populations of *D. odorifera.* The analysis revealed 7 alleles for CCRS2, as well as 13 SSR genotypes in a sample of 105 individuals. SSR markers with a high degree of polymorphism and stability are crucial tools for analyzing genetic relationships. CCRS2 was discovered to be highly polymorphic, with PIC values greater than 0.50, suggesting that those alleles were present in more than 50% of the germplasm. PIC values may be affected by a variety of variables, including sampling techniques, the quantity of SSRs, and the kinds of SSR motif repeats (Liu et al., 2019b). Highly polymorphic SSR markers are helpful for assessing genetic variety, but utilizing only the most polymorphic markers could bias the overall genetic diversity (Liu et al., 2019b), especially in conservation studies (Väli et al., 2008). Furthermore, a single SSR marker was not able to completely distinguish all individuals of the natural population, making it difficult to further analysis its population structure. Therefore, more SSR markers need to be developed in subsequent studies to fully evaluate the genetic relationship between *D. odorifera* populations.

## Conclusion

In summary, a total of 24 CCR genes were identified from *D. odorifera* genome which located on 10 different chromosomes. Phylogenetic analysis revealed that seven DoCCRs were clustered with functional CCRs of dicotyledons involved in lignin biosynthesis. RNA-seq data analysis showed different expression patterns of *DoCCRs* between different tissues and organs, and that some genes were induced by wounding stress. 74 SSRs were identified in 19 *DoCCR* genes, located in introns or UTRs and dominated by mononucleotides. A pair of SSR primers with high polymorphism were designed and screened based on candidate *DoCCRs*. These results provide useful information for the functional studies of *DoCCRs* and future genetic improvements in the wood properties and environmental resistance of *D. odorifera.*

## Data availability statement

The original contributions presented in the study are included in the article/**Supplementary Material**. Further inquiries can be directed to the corresponding author.

## Author contributions

JC designed the research. YW, JX, WZ, JL and JC performed the research. All authors analyzed and interpreted the data. JC and YW wrote the paper. All authors commented on the manuscript.
