# Supplementary material for: Genome-wide identification, characterization, and genetic diversity of CCR gene family in Dalbergia odorifera
Source: Front Plant Sci. 2022 Dec 19;13:1064262. doi: 10.3389/fpls.2022.1064262 (PMC9806228; doi:10.3389/fpls.2022.1064262)
Supplement: Supplementary file 1 [file Image_1.pdf]

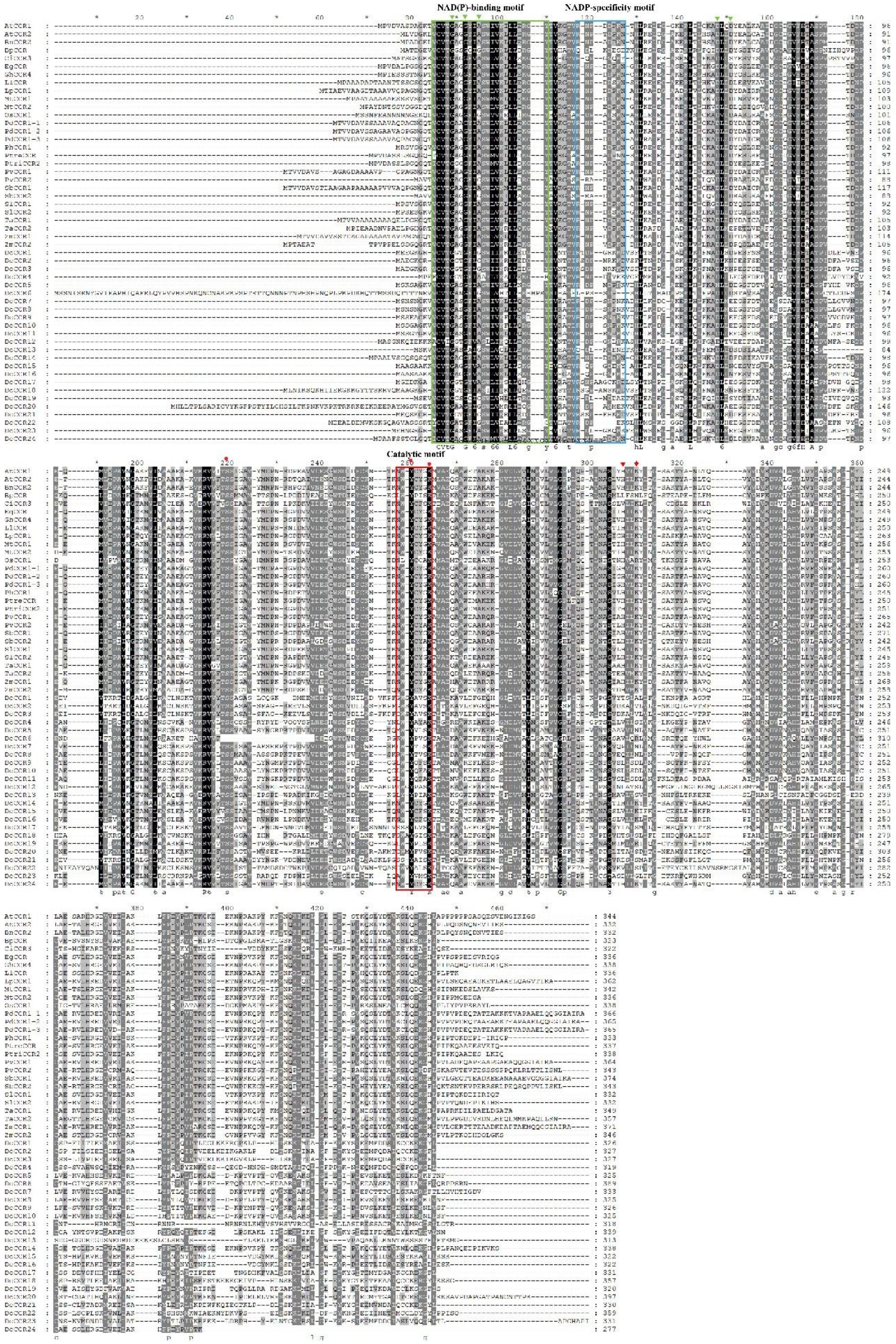

Figure S1. Multiple sequence alignments of the deduced amino acid sequences of DoCCRs with CCRs from other plant species. The amino acid sequences were alingned using MUSCLE. The shaded amino acids denoted identical or similar amino

acids. The green and blue box indicate NAD(P)-binding and NADP specificity motif, respectively. The red box indicates catalytic motif. Green inverted triangle indicates motif $G(X)_2G(X)_2A$ and $D(X)_2D$. The catalytic triad Ser-Tyr-Lys is indicated by red circles. Red inverted triangle indicates a novel motif $H(X)_2K$ (CCR-SBM or CCR substrate binding motif). At, *A. thaliana*; Bp, *B. platyphylla*; Bn, *B. napus*; Ci, *C. intermedia*; Do, *D. odorifera*; Eg, *E. gunnii*; Gm, *G. mexicanum*; Ll, *L. leucocephala*; Lp, *L. perenne*; Mt, *M. truncatula*; Os, *O. sativa*; Pv, *P. virgatum*; Pd, *P. dilatatum*; Ph, *P. hybrida*; Ptre, *P. tremuloides*; Ptri, *P. trichocarpa*; Sl, *S. lycopersicum*; Sb, *S. bicolor*; Ta, *T. aestivum*; Zm, *Z. mays*. Detailed information of all CCRs from other plants are showed in Supplemental Table S1.

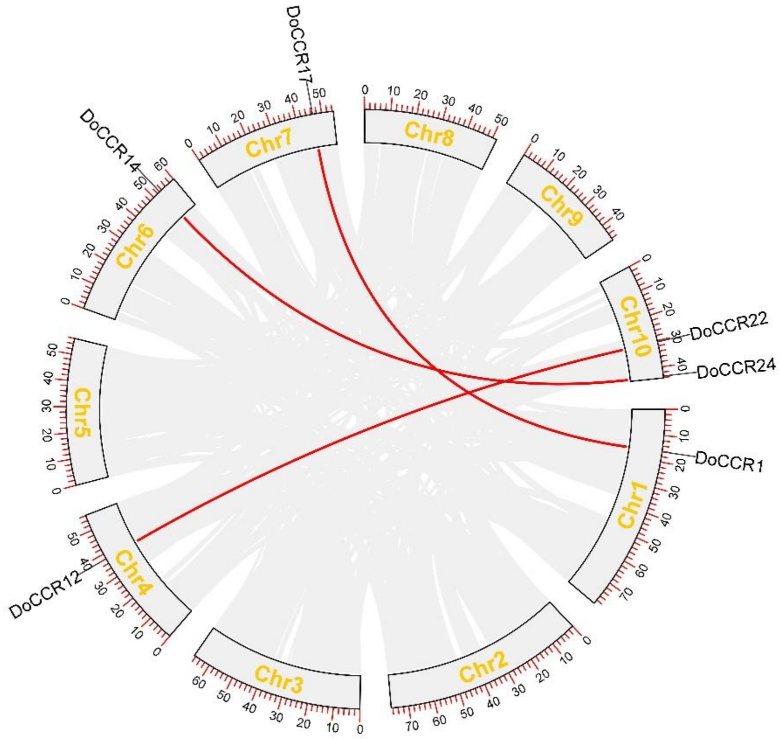

Figure S2. Syntenic relationships among *CCR* genes in *D. odorifera*. Red lines in highlight indicate the syntenic *DoCCR* gene pairs.

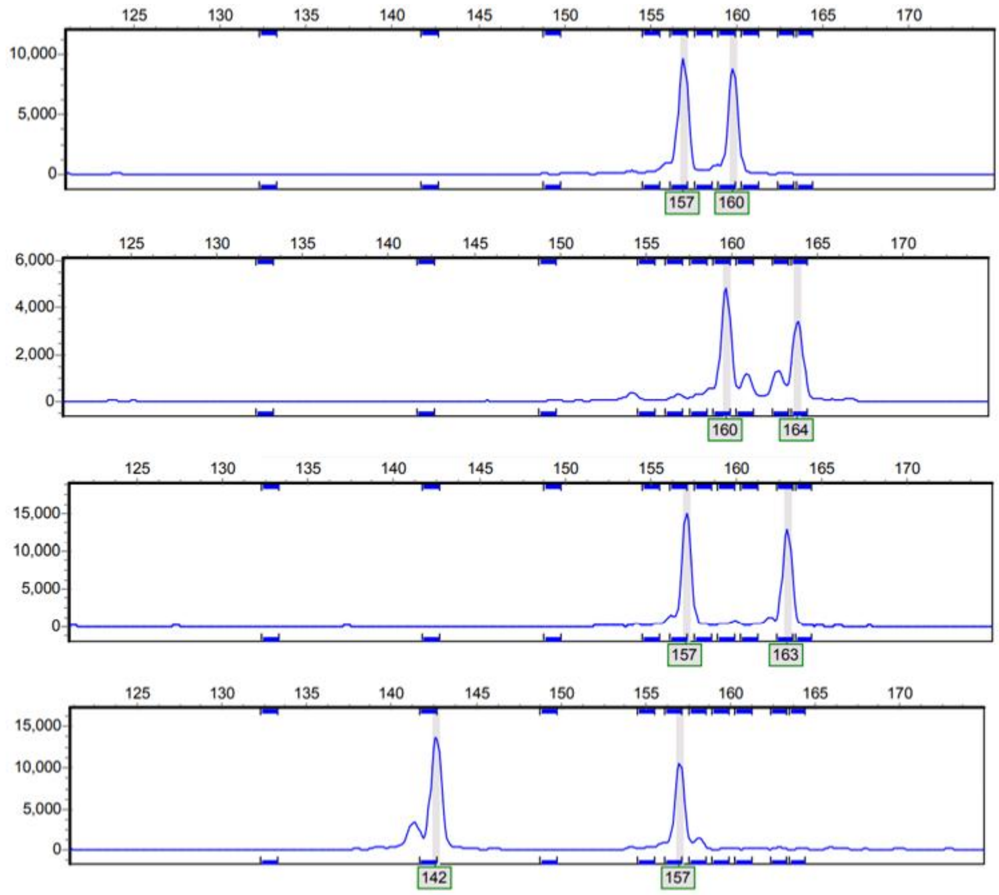

Figure S3. Amplification results of CCRS2 locus in four *D. odorifera* samples.

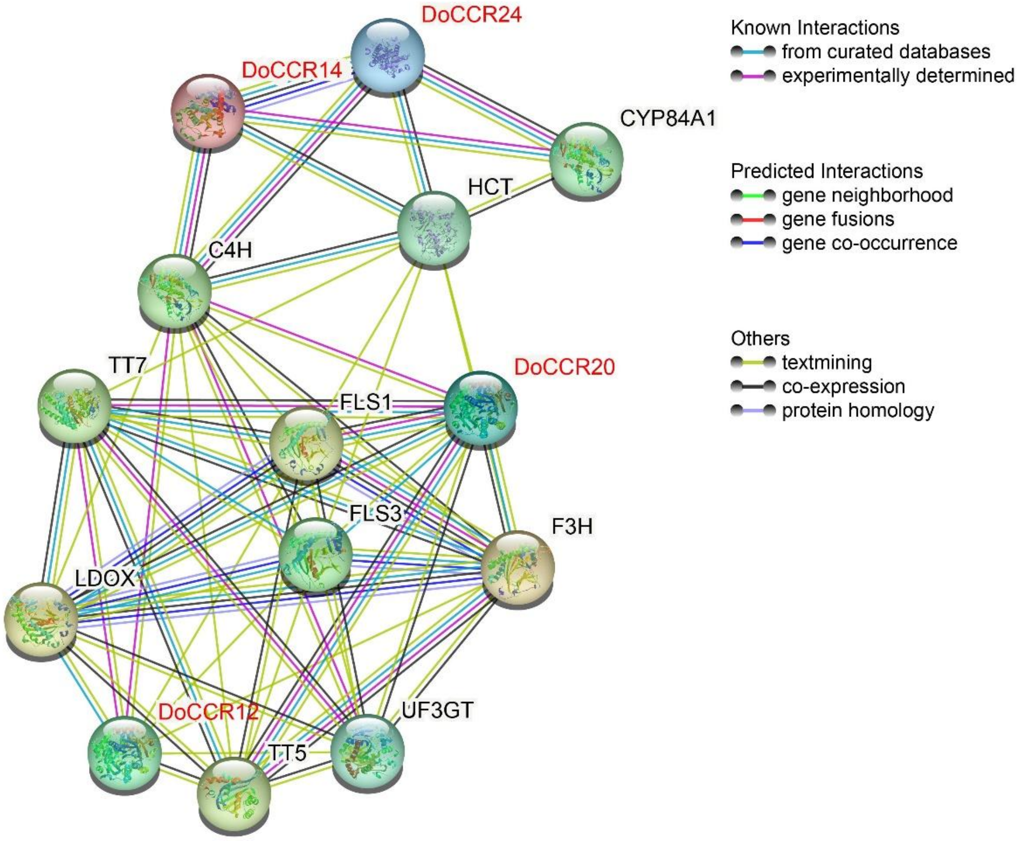

Figure S4. Interaction network analysis of DoCCR proteins using *Arabidopsis* orthologs. Colored lines between the proteins indicate the various types of interaction evidence.
