# Supplementary material for: Genome-wide identification, characterization, and genetic diversity of CCR gene family in Dalbergia odorifera
Source: Front Plant Sci. 2022 Dec 19;13:1064262. doi: 10.3389/fpls.2022.1064262 (PMC9806228; doi:10.3389/fpls.2022.1064262)
Supplement: Supplementary file 2 [file Table_1.pdf]

Table S1. The sources of 28 functional CCR proteins from 19 plant species.

Taxnomy	Species	Gene Name	Uniprot Entry	Reference
Monocotyledons	Arabidopsis thaliana	AtCCR1	Q9S9N9	Lauvergeat et al., 2001
		AtCCR2	Q9SAH9	
	Brassica napus	BnCCR	A0A078HQM8	Liu et al., 2021
	Betula platyphylla	BpCCR	I6WM26	Zhang et al., 2012
	Caragana intermedia	CiCCR3	A0A344X2B3	
	Eucalyptus gunnii	EgCCR	O04877	Lacombe et al., 1997; Piquemal et al., 1998
	Gossypium mexicanum	GhCCR4	E3NZI6	
	Leucaena leucocephala	LlCCR	Q08GL0	Prashant et al., 2011
	Lolium perenne	LpCCR1	Q8VWI9	McInnes et al., 2002; Tu et al., 2010
	Medicago truncatula	MtCCR1	A0A072VDF2	Jackson et al., 2008; Zhou et al., 2010
		MtCCR2	G7JEE5	
	Petunia hybrida	PhCCR1	A0A059TC02	Pan et al., 2014; Muhlemann et al., 2014
	Populus tremuloides	PtreCCR	Q9M631	Li et al., 2005
	Populus trichocarpa	PtriCCR2	O65880	Leplé et al., 2007; Van et al., 2014
	Solanum lycopersicum	SlCCR1	Q4U1I5	van der Rest et al, 2006
		SlCCR2	Q4U1I4	
Dicotyledons	Oryza sativa	OsCCR1	Q6K9A2	Kawasaki et al., 2006
	Panicum virgatum	PvCCR1	D2IX40	Escamilla-Treviño et al., 2010
		PvCCR2	D2IX45	
	Paspalum dilatatum	PdCCR1-1	S5RYC4	Giordano et al., 2014
		PdCCR1-2	S5RCD3	
		PdCCR1-3	S5RGZ3	
	Sorghum bicolor	SbCCR1	C5YLL4	Li et al, 2016; Sattler et al., 2017
		SbCCR2	C5XWV7	
	Triticum aestivum	TaCCR1	A8DNN6	Ma and Tian, 2005; Ma, 2007
		TaCCR2	Q4KUK8	
	Zea mays	ZmCCR1	O24563	Pichon et al., 1998; Tamasloukht et al., 2011
		ZmCCR2	O82726	

Table S2. Geographical location of 105 *D. odorifera* samples.

Population	Location	Size	Code	E longitude	N latitude
HK	Haikou	28	A001-028	110°13′27″-110°32′06″	19°42′27″-20°02′23″
DZ	Danzhou	14	A029-042	109°13′28″-109°40′15″	19°27′59″-19°47′34″
DF	Dongfang	6	A043-048	108°40′36″-108°47′40″	18°48′11″-19°06′59″
LD	Ledong	9	A049-057	108°42′39″-108°57′35″	18°27′34″-18°41′46″
SY	Sanya	14	A058-071	109°09′43″-109°32′56″	18°13′34″-18°22′35″
WC	Wenchang	3	A072-074	110°44′48″-110°54′51″	19°32′37″-19°34′56″
WN	Wanning	8	A075-082	110°13′12″-110°29′02″	18°47′52″-19°01′16″
CM	Chengmai	3	A083-085	110°00′50″-110°06′41″	19°44′41″-19°55′37″
BT	Baoting	2	A086-087	109°42′42″-109°48′40″	18°38′41″-18°39′18″
QH	Qionghai	2	A088-089	110°24′23″-110°28′58″	19°07′56″-19°15′34″
LG	Lingao	6	A090-095	109°33′50″-109°41′00″	19°54′43″-19°56′44″
TC	Tunchang	3	A096-098	109°59′04″-110°06′36″	19°13′14″-19°21′26″
DA	Dingan	2	A099-100	110°12′03″-110°14′29″	19°36′12″-19°38′56″
LS	Lingshui	1	A101	110°02′38″	18°30′44.39″
BS	Baisha	2	A102-103	109°16′38″-109°26′57″	19°09′39″-19°13′40″
CJ	Changjiang	1	A104	109°05′23″	19°07′39″
WZS	Wuzhishan	1	A105	110°23′26″	19°59′26″

Table S3. Physicochemical properties of functional CCR proteins in other plants.

Gene name	Protein Length (aa)	Molecular Weight (kDa)	Theoretical pI	Instability Index	Grand Average of Hydropathicity
AtCCR1	344	37.49	6.13	32.17	-0.260
AtCCR2	332	36.62	6.40	25.89	-0.166
BnCCR	332	36.57	6.26	31.35	-0.202
BpCCR	323	35.36	5.35	29.28	0.029
CiCCR3	322	35.81	6.62	36.24	-0.170
EgCCR	336	36.56	5.87	31.72	-0.098
GhCCR4	338	37.17	6.33	36.07	-0.196
LlCCR	336	36.52	6.32	36.09	-0.154
LpCCR1	362	39.13	5.68	32.94	-0.101
MtCCR1	342	37.11	6.52	31.65	-0.117
MtCCR2	336	37.20	6.13	31.01	-0.266
OsCCR1	338	37.39	8.27	29.23	-0.209
PdCCR1-1	366	39.62	6.20	33.41	-0.111
PdCCR1-2	365	39.49	6.02	32.09	-0.127
PdCCR1-3	365	39.49	6.27	32.01	-0.128
PhCCR1	333	36.89	5.94	28.70	-0.214
PtreCCR	337	37.06	5.87	33.93	-0.193
PtriCCR2	338	37.11	5.88	36.04	-0.204
PvCCR1	364	39.04	5.80	31.74	-0.069
PvCCR2	343	37.88	6.32	34.57	-0.218
SbCCR1	374	40.23	5.51	31.18	-0.095
SbCCR2	343	37.89	8.85	42.50	-0.217
SlCCR1	332	36.79	6.66	29.90	-0.180
SlCCR2	332	36.82	7.07	30.25	-0.210
TaCCR1	349	37.37	6.07	31.79	-0.087
TaCCR2	357	39.48	6.52	34.28	-0.219
ZmCCR1	371	40.11	6.27	33.23	-0.126
ZmCCR2	346	38.01	6.31	31.68	-0.192

Table S4. Sequence similarities between CCR proteins.

	LpCCR1	OsCCR1	SlCCR1	SlCCR2	EgCCR	PtreCCR	PvCCR1	PvCCR2	AtCCR1	AtCCR2	TaCCR1	TaCCR2	ZmCCR1	ZmCCR2	BnCCR2	LlCCR	MtCCR1	MtCCR2	PtriCCR2	BpCCR	GhCCR4	PdCCR1-1	PdCCR1-2	PdCCR1-3	CiCCR3	PhCCR1	SbCCR1	SbCCR2
DoCCR1	50.80	45.89	54.18	53.18	52.54	54.11	50.40	47.38	52.35	54.05	52.49	47.35	48.70	49.72	52.62	53.31	52.10	50.72	55.40	53.96	52.15	50.13	50.53	50.13	52.06	53.45	49.35	46.15
DoCCR2	48.27	45.79	50.70	52.17	52.51	52.11	48.68	48.22	52.51	53.87	49.72	49.19	48.06	49.72	52.72	51.69	50.56	51.27	52.54	55.19	51.86	48.28	48.15	48.28	52.79	50.00	47.29	47.40
DoCCR3	48.28	46.20	50.86	53.01	52.84	53.28	48.41	48.21	51.52	53.30	49.73	49.46	47.01	49.45	52.15	51.43	50.99	51.43	53.71	53.78	51.58	48.29	48.16	48.43	52.79	50.43	47.30	47.12
DoCCR4	50.14	48.71	56.65	56.60	55.94	55.62	50.27	48.74	53.24	54.87	51.40	49.59	47.27	51.40	53.67	53.85	54.11	55.94	56.32	55.49	56.00	48.42	48.66	49.06	55.59	57.02	47.16	48.73
DoCCR5	61.14	56.65	64.90	63.56	63.56	62.21	61.08	55.37	61.82	61.18	62.82	56.32	59.95	58.07	62.35	61.81	62.18	63.37	62.61	66.77	63.77	60.75	59.84	60.22	59.88	65.00	59.74	55.65
DoCCR6	38.22	43.66	43.06	41.87	42.24	43.68	39.51	38.62	39.91	40.80	41.15	39.82	38.68	42.69	42.45	43.34	43.88	44.15	43.57	48.18	42.86	39.50	39.82	39.73	41.12	42.72	38.65	39.13
DoCCR7	58.18	57.26	62.79	61.85	59.77	60.29	57.33	52.38	58.01	58.55	58.71	55.31	56.54	58.03	61.00	57.59	59.09	59.65	60.12	61.31	62.43	57.10	56.99	56.30	57.52	62.97	56.36	52.66
DoCCR8	58.81	57.64	62.65	61.34	61.05	60.00	58.49	52.68	60.23	60.41	60.11	56.16	56.73	58.03	61.29	57.85	58.57	58.55	60.12	63.41	61.56	57.10	57.26	56.57	59.82	63.05	56.51	52.68
DoCCR9	59.19	58.91	62.06	61.34	61.16	60.87	58.33	52.11	60.23	59.82	59.94	56.71	56.88	56.78	61.88	59.01	60.29	61.74	60.69	65.85	60.69	57.64	57.91	57.22	59.27	61.88	56.43	50.70
DoCCR10	60.60	58.38	63.72	64.14	62.39	61.45	60.00	52.82	60.97	60.59	60.85	57.42	59.15	58.92	61.76	60.64	60.74	61.63	62.03	64.63	62.03	58.87	58.76	58.06	59.57	63.53	58.42	52.54
DoCCR11	53.85	42.46	52.96	48.40	49.19	52.49	52.01	41.41	51.96	42.13	52.63	49.73	52.09	46.06	46.51	50.97	50.55	50.82	50.82	52.69	53.20	52.14	51.74	52.28	49.31	45.43	51.17	41.58
DoCCR12	49.23	46.47	50.42	51.26	51.25	51.10	48.46	45.16	50.55	52.66	49.34	48.14	46.60	49.46	50.70	52.19	50.56	51.12	52.35	54.83	51.54	48.21	47.57	47.59	55.39	50.84	46.87	44.89
DoCCR13	44.73	42.90	47.34	46.83	43.58	45.95	43.04	40.26	43.92	44.86	46.38	41.09	41.98	38.75	40.21	46.17	45.97	44.99	46.47	60.56	44.72	41.75	42.36	48.14	46.09	46.26	44.61	42.55
DoCCR14	74.31	69.68	88.17	86.98	88.46	87.98	75.55	70.82	82.37	83.82	76.50	75.00	74.39	78.16	83.48	86.38	88.92	89.47	87.61	61.05	89.12	74.32	73.97	74.04	58.67	86.73	73.80	70.82
DoCCR15	57.18	57.51	61.65	61.65	60.64	60.47	56.87	53.95	58.12	60.00	60.28	56.95	55.70	58.36	60.88	60.93	59.77	60.06	60.00	59.88	60.87	55.91	57.14	56.18	90.40	61.76	56.58	54.24
DoCCR16	56.37	56.07	61.36	61.36	59.77	59.59	56.06	52.82	57.55	59.24	59.72	55.86	54.91	56.94	59.12	60.06	58.62	59.77	59.13	58.38	59.71	55.91	56.33	56.18	90.71	61.47	55.79	53.39
DoCCR17	47.49	47.21	51.00	51.71	50.84	51.83	46.72	46.70	49.72	51.01	47.81	47.86	45.38	48.77	51.59	49.15	49.59	50.99	51.54	56.12	50.99	45.99	45.76	46.25	50.58	50.14	44.97	46.43
DoCCR18	51.55	45.19	49.87	50.67	50.67	51.06	52.32	43.08	49.87	51.74	50.65	46.98	51.29	48.70	50.94	51.09	52.70	50.13	51.72	48.78	52.01	51.15	49.24	51.41	49.20	49.73	52.30	42.82
DoCCR19	55.71	50.99	60.36	61.00	60.82	58.60	56.22	54.73	56.86	57.99	57.18	54.67	54.38	55.34	58.28	58.48	57.79	59.06	59.30	86.07	59.30	55.38	56.06	55.38	58.05	60.47	54.74	54.73
DoCCR20	50.24	45.63	51.34	51.72	53.06	52.96	49.54	47.53	50.61	50.00	49.17	51.08	48.40	52.33	50.74	49.76	53.09	49.52	51.45	47.00	51.20	49.77	49.65	49.19	47.76	52.59	47.96	47.74
DoCCR21	47.16	46.54	51.55	52.11	50.41	50.83	47.18	45.50	49.86	50.42	48.92	47.18	47.41	47.70	50.14	51.29	48.22	50.14	50.97	53.71	51.52	48.82	48.44	47.03	49.01	51.12	47.56	44.65
DoCCR22	48.59	46.68	49.47	48.28	51.34	49.47	48.46	42.71	48.69	48.13	48.29	46.82	47.86	47.79	47.72	49.33	48.28	50.54	49.87	46.40	50.79	48.21	47.85	48.08	47.28	50.00	47.37	42.18
DoCCR23	53.26	52.60	54.18	56.05	54.13	54.70	53.24	50.00	51.84	52.74	54.17	53.02	49.61	54.39	54.25	52.42	53.24	54.47	55.11	58.60	55.20	51.72	51.33	51.46	56.27	53.60	50.65	50.28
DoCCR24	63.54	59.29	72.11	70.92	72.70	73.67	63.41	60.23	70.43	69.23	65.33	61.84	62.26	65.90	69.82	77.98	76.61	75.30	73.67	54.10	73.45	63.11	63.84	62.84	53.82	70.71	62.57	59.38

Table S5. Ka, Ks and Ka/Ks of replication pairs of *DoCCRs*.

Gene Name	Gene Name	Duplication type	Ka	Ks	Ka/Ks
DoCCR1	DoCCR2	Tandem duplication	0.140727	0.615468	0.22865
DoCCR2	DoCCR3	Tandem duplication	0.026942	0.0729515	0.369313
DoCCR7	DoCCR8	Tandem duplication	0.0842733	0.27465	0.306838
DoCCR8	DoCCR9	Tandem duplication	0.197238	0.508429	0.387937
DoCCR9	DoCCR10	Tandem duplication	0.063124	0.228876	0.2758
DoCCR10	DoCCR11	Tandem duplication	0.217544	0.378383	0.574931
DoCCR1	DoCCR17	Segmental duplication	0.346644	2.00078	0.173255
DoCCR22	DoCCR12	Segmental duplication	0.59811	2.15408	0.277663
DoCCR24	DoCCR14	Segmental duplication	0.090619	0.615493	0.14723

Table S6. Orthologous relationships between *DoCCR* genes in *D.odorifera* with other *CCR* genes in *A. thaliana*, *M. truncatula*, *O. sativa* and *Z. mays.*

Chromosome	Gene Name	Chromosome	Gene ID
Do-1	DoCCR1	At-2	AT2G45400.1
Do-3	DoCCR7	At-1	AT1G51410.1
Do-4	DoCCR12	At-1	AT1G61720.1
Do-6	DoCCR14	At-1	AT1G15950.1
Do-6	DoCCR14	At-1	AT1G80820.1
Do-7	DoCCR17	At-2	AT2G45400.1
Do-8	DoCCR19	At-5	AT5G58490.1
Do-10	DoCCR22	At-4	AT4G27250.1
Do-10	DoCCR24	At-1	AT1G80820.1
Do-10	DoCCR24	At-1	AT1G15950.1
Do-1	DoCCR1	Mt-7	Medtr7g074710.1
Do-2	DoCCR5	Mt-3	Medtr3g005170.1
Do-3	DoCCR6	Mt-2	Medtr2g028620.1
Do-3	DoCCR7	Mt-4	Medtr4g077100.1
Do-4	DoCCR12	Mt-4	Medtr4g092080.1
Do-5	DoCCR13	Mt-3	Medtr3g031650.3
Do-5	DoCCR13	Mt-5	Medtr5g072620.1
Do-6	DoCCR14	Mt-2	Medtr2g104960.1
Do-6	DoCCR14	Mt-4	Medtr4g006940.1
Do-7	DoCCR17	Mt-4	Medtr4g023730.1
Do-7	DoCCR18	Mt-7	Medtr7g074710.1
Do-8	DoCCR19	Mt-3	Medtr3g031650.3
Do-8	DoCCR19	Mt-5	Medtr5g072620.1
Do-8	DoCCR20	Mt-1	Medtr1g022440.1
Do-10	DoCCR22	Mt-4	Medtr4g081440.1
Do-10	DoCCR23	Mt-2	Medtr2g101330.1
Do-10	DoCCR24	Mt-2	Medtr2g104960.1
Do-10	DoCCR24	Mt-4	Medtr4g006940.1
Do-8	DoCCR19	Os-1	LOC_Os01g61230.1
Do-10	DoCCR22	Os-4	LOC_Os04g53780.1
Do-8	DoCCR19	Zm-3	Zm00001d043013_T001

Table S7. All SSR loci in *DoCCR* genes.

Gene Name	Start	End	SSR	Type	Size	Length	Location
DoCCR1	210	220	(T)11	Mononucleotide	11	11	Intron
DoCCR1	236	246	(T)11	Mononucleotide	11	11	Intron
DoCCR1	2816	2825	(T)10	Mononucleotide	10	10	Intron
DoCCR1	3485	3494	(T)10	Mononucleotide	10	10	3’UTR
DoCCR2	316	337	(TA)11	Dinucleotide	11	22	Intron
DoCCR2	2011	2044	(TA)17	Dinucleotide	17	34	Intron
DoCCR2	3238	3250	(T)13	Mononucleotide	13	13	Intron
DoCCR3	96	112	(C)17	Mononucleotide	17	17	5’UTR
DoCCR3	1551	1561	(T)11	Mononucleotide	11	11	Intron
DoCCR3	2324	2337	(TA)7	Dinucleotide	7	14	Intron
DoCCR3	2766	2777	(TA)6	Dinucleotide	6	12	Intron
DoCCR4	974	989	(T)16	Mononucleotide	16	16	Intron
DoCCR4	3367	3378	(A)12	Mononucleotide	12	12	3’UTR
DoCCR5	303	327	(TTTTA)5	Pentanucleotide	5	25	Intron
DoCCR5	757	777	(TAT)7	Trinucleotide	7	21	Intron
DoCCR5	1498	1514	(T)17	Mononucleotide	17	17	Intron
DoCCR5	1633	1646	(T)14	Mononucleotide	14	14	Intron
DoCCR5	9605	9615	(T)11	Mononucleotide	11	11	Intron
DoCCR5	10679	10690	(T)12	Mononucleotide	12	12	Intron
DoCCR5	12194	12208	(T)15	Mononucleotide	15	15	Intron
DoCCR6	1020	1029	(T)10	Mononucleotide	10	10	Intron
DoCCR6	1432	1442	(T)11	Mononucleotide	11	11	Intron
DoCCR7	107	119	(T)13	Mononucleotide	13	13	5’UTR
DoCCR7	851	872	(C)22	Mononucleotide	22	22	Intron
DoCCR7	3277	3297	(TTA)7	Trinucleotide	7	21	Intron
DoCCR8	3170	3179	(T)10	Mononucleotide	10	10	Intron
DoCCR8	3203	3212	(T)10	Mononucleotide	10	10	Intron
DoCCR8	3761	3774	(T)14	Mononucleotide	14	14	Intron
DoCCR8	4163	4172	(T)10	Mononucleotide	10	10	Intron
DoCCR9	2758	2772	(TTC)5	Trinucleotide	5	15	Intron
DoCCR11	171	188	(TGT)6	Trinucleotide	6	18	Intron
DoCCR11	193	202	(T)10	Mononucleotide	10	10	Intron
DoCCR11	2107	2118	(TG)6	Dinucleotide	6	12	Intron
DoCCR11	6491	6503	(T)13	Mononucleotide	13	13	Intron
DoCCR12	373	383	(A)11	Mononucleotide	11	11	Intron
DoCCR12	960	972	(A)13	Mononucleotide	13	13	Intron
DoCCR12	1425	1434	(A)10	Mononucleotide	10	10	Intron
DoCCR12	3312	3325	(T)14	Mononucleotide	14	14	Intron
DoCCR13	2136	2146	(A)11	Mononucleotide	11	11	Intron
DoCCR13	2972	2986	(ATA)5	Trinucleotide	5	15	Intron
DoCCR13	4214	4225	(T)12	Mononucleotide	12	12	Intron

DoCCR13	4548	4561	(T)14	Mononucleotide	14	14	Intron
DoCCR13	5489	5498	(A)10	Mononucleotide	10	10	Intron
DoCCR14	46	69	(TC)12	Dinucleotide	12	24	5'UTR
DoCCR14	2003	2020	(TA)9	Dinucleotide	9	18	Intron
DoCCR14	3183	3195	(T)13	Mononucleotide	13	13	Intron
DoCCR14	4460	4470	(T)11	Mononucleotide	11	11	Intron
DoCCR15	617	628	(T)12	Mononucleotide	12	12	Intron
DoCCR15	4828	4837	(T)10	Mononucleotide	10	10	Intron
DoCCR16	252	269	(GAA)6	Trinucleotide	6	18	5'UTR
DoCCR16	1767	1778	(T)12	Mononucleotide	12	12	Intron
DoCCR17	164	175	(AT)6	Dinucleotide	6	12	5'UTR
DoCCR17	542	552	(T)11	Mononucleotide	11	11	Intron
DoCCR17	842	851	(T)10	Mononucleotide	10	10	Intron
DoCCR17	4812	4823	(A)12	Mononucleotide	12	12	Intron
DoCCR17	5429	5438	(A)10	Mononucleotide	10	10	Intron
DoCCR18	2173	2182	(T)10	Mononucleotide	10	10	Intron
DoCCR19	233	247	(TTC)5	Trinucleotide	5	15	Intron
DoCCR19	762	775	(TC)7	Dinucleotide	7	14	Intron
DoCCR19	773	791	(TCT)6	Trinucleotide	6	18	Intron
DoCCR19	4947	4957	(T)11	Mononucleotide	11	11	Intron
DoCCR19	5456	5466	(G)11	Mononucleotide	11	11	Intron
DoCCR19	7175	7185	(G)11	Mononucleotide	11	11	Intron
DoCCR20	1238	1252	(T)15	Mononucleotide	15	15	Intron
DoCCR20	1851	1861	(T)11	Mononucleotide	11	11	Intron
DoCCR20	1908	1919	(A)12	Mononucleotide	12	12	Intron
DoCCR20	2097	2108	(T)12	Mononucleotide	12	12	Intron
DoCCR20	2731	2770	(AT)20	Dinucleotide	20	40	Intron
DoCCR20	4130	4167	(TA)19	Dinucleotide	19	38	Intron
DoCCR22	949	963	(T)15	Mononucleotide	15	15	Intron
DoCCR22	3245	3255	(T)11	Mononucleotide	11	11	Intron
DoCCR23	1198	1209	(AC)6	Dinucleotide	6	12	Intron
DoCCR24	286	319	(TC)17	Dinucleotide	17	34	Intron
DoCCR24	831	840	(A)10	Mononucleotide	10	10	Intron

Table S8. The type and number of SSR repeat motifs in *DoCCRs*.

Repeat type	Number of motif type	Repeat motif	Number	Percentage
Mononucleotide	2	A/T	48	64.86%
		C/G	4	5.41%
Dinucleotide	3	AC/GT	2	2.70%
		AG/CT	3	4.05%
		AT/AT	8	10.81%
Trinucleotide	3	AAC/GTT	1	1.35%
		AAG/CTT	4	5.41%
		AAT/ATT	3	4.05%
Pentanucleotide	1	AAAAT/ATTTT	1	1.35%
Total	9		74	100.00%

Table S9. The information of primers of SSR markers in *DoCCR5*.

Locus	Repeat motif	Primer (5'-3')	Expected size (bp)	Gene
CCRS2	(TAT)7	F ATTGGAGTTGCGCGTTGTTC<br>R GGGTTCCTTTCACTGCTGGA	135	DoCCR5

Table S10. Polymorphism characteristics of one SSR loci in 105 *D. odorifera* samples.

Locus	N	Na	Ne	I	Ho	He	F	PIC
CCRS2	105	7.000	2.669	1.248	0.610	0.625	0.025	0.586

Abbreviations: *N*: number of effective individuals; *Na*: number of alleles; *Ne*: number of effective alleles; *I*: Shannon's information index; *Ho*: observed heterozygosity; *He*: expected heterozygosity; *F*: Fixed index; PIC: polymorphism information content index.

Table S11. Genetic diversity of the various source populations of *D. odorifera.*

Population	N	Na	Ne	I	Ho	He	F
HK	28	5.000	2.810	1.255	0.679	0.644	-0.053
DZ	14	4.000	2.320	1.061	0.643	0.569	-0.130
SY	14	4.000	2.780	1.152	0.643	0.640	-0.004
LD	9	4.000	2.492	1.087	0.667	0.599	-0.113
DF	6	4.000	2.400	1.075	0.833	0.583	-0.429
WN	8	5.000	2.560	1.190	0.625	0.609	-0.026
LG	6	3.000	2.000	0.868	0.500	0.500	0.000
Mean	12.143	4.143	2.480	1.098	0.656	0.592	-0.108

Abbreviations: *N*: number of effective individuals; *Na*: number of alleles; *Ne*: number of effective alleles; *I*: Shannon's information index; *Ho*: observed heterozygosity; *He*: expected heterozygosity; *F*: Fixed index.
